# Therapeutic Advances in Major NBIA Disorders: Current Strategies and Translational Challenges

**DOI:** 10.3390/neurolint18070133

**Published:** 2026-07-10

**Authors:** Floriana Cascone, Gemma Gasparini, Valeria Tiranti, Ivano Di Meo

**Affiliations:** Unit of Medical Genetics and Neurogenetics, Fondazione IRCCS Istituto Neurologico Carlo Besta, 20126 Milan, Italy; floriana.cascone@istituto-besta.it (F.C.); gemma.gasparini@istituto-besta.it (G.G.); valeria.tiranti@istituto-besta.it (V.T.)

**Keywords:** PKAN, CoPAN, PLAN, BPAN, MPAN, Neurodegeneration with brain iron accumulation, lipid metabolism, autophagy, CoA biosynthesis

## Abstract

Neurodegeneration with brain iron accumulation (NBIA) comprises a group of rare genetic movement disorders characterized by progressive neurological deterioration, dystonia, parkinsonism, spasticity, and abnormal iron deposition in the basal ganglia. Although iron accumulation is the shared neuroradiological hallmark, most NBIA genes do not directly regulate iron metabolism. Instead, major NBIA forms arise from disruption of distinct but converging cellular pathways, including coenzyme A (CoA) biosynthesis, lipid metabolism, mitochondrial function, and autophagy. This narrative review aims to examine the pathogenic mechanisms of major NBIA disorders, namely pantothenate kinase-associated neurodegeneration (PKAN), COASY protein-associated neurodegeneration (CoPAN), PLA2G6-associated neurodegeneration (PLAN), mitochondrial membrane protein-associated neurodegeneration (MPAN), and beta-propeller protein-associated neurodegeneration (BPAN), and how these insights are guiding therapeutic development. Preclinical strategies aimed at restoring CoA metabolism, improving mitochondrial function, limiting lipid peroxidation, modulating autophagy, or correcting the underlying genetic defect have shown encouraging results, although none have yet reached robust clinical validation. Clinical translation remains limited by disease rarity, clinical heterogeneity, absence of validated biomarkers, and preclinical models that only partially recapitulate human pathology. Advancing the field will depend on earlier molecular diagnosis, biomarkers capable of tracking disease stage, and trial designs suited to ultra-rare populations. NBIA thus offers a paradigm for how mechanistic classification of a genetically defined disease group can redirect therapeutic strategy away from a shared radiological feature and toward pathway-specific intervention.

## 1. Introduction

Neurodegeneration with brain iron accumulation (NBIA) disorders represent a clinically and genetically heterogeneous group of rare neurodegenerative diseases. Their shared radiological feature, abnormal iron deposition in deep brain nuclei, has historically guided diagnosis and nomenclature [1]. However, genetic discoveries have shown that most NBIA forms arise from defects in pathways not primarily devoted to iron metabolism [2]. This has shifted the field from an iron-centered view toward a broader mechanistic framework that integrates mitochondrial dysfunction, lipid dysregulation, impaired autophagy, vesicle trafficking defects, and altered coenzyme A (CoA) metabolism.

In this narrative review, we discuss disease pathomechanisms and emerging therapeutic strategies for major NBIA forms and identify the key gaps that should be addressed to advance mechanism-based and precision-oriented therapies.

## 2. Neurodegeneration with Brain Iron Accumulation (NBIA): Genetic and Clinical Overview

NBIA disorders comprise multiple rare, monogenic neurodegenerative disorders that differ in inheritance, age at onset, neurological presentation, and disease course. To date, advances in genetic research have identified 18 NBIA-associated genes (Table 1), which encode proteins involved in distinct, although partly overlapping, cellular pathways.

Only three genes are directly linked to iron homeostasis: ceruloplasmin (*CP*), ferritin light chain (*FTL*), and ferritin heavy chain (*FTH1*) [5,21]. This observation supports a mechanistic classification of NBIA disorders according to the primarily affected pathways. Besides iron homeostasis, these include coenzyme A (CoA) biosynthesis (*PANK2* and *COASY*), lipid metabolism (*PLA2G6*, *FA2H*, *C19orf12*, *SCP2*, *CRAT*, *SLC27A3*, *MECR*), autophagy and lysosomal homeostasis (*WDR45*, *ATP13A2*, *AP4M1*, *REPS1*), and genes with still incompletely understood functions (*DCAF17* and *GTPBP2*) [11,13] (Table 1).

Recent genetic and clinical studies have further expanded the NBIA spectrum by identifying new disease-associated genes and refining the phenotypic boundaries of previously recognized disorders [5,11,13,18,20,22,23,24,25,26,27,28,29,30]. These advances underscore that NBIA is not a single mechanistic entity, but a genetically defined group in which overlapping neuroradiological features arise from distinct cellular defects [2].

The overall estimated prevalence of NBIA is 1–3 per 1,000,000 individuals, although estimates vary substantially across subtypes, populations, and geographic regions [11]. Pantothenate kinase-associated neurodegeneration (PKAN), β-propeller-associated neurodegeneration (BPAN), mitochondrial membrane protein-associated neurodegeneration (MPAN), and PLA2G6-associated neurodegeneration (PLAN) are among the most frequent forms within this spectrum [1]. Moreover, population-genetic studies further suggest that, among autosomal recessive NBIA disorders, variants in *PLA2G6*, *PANK2*, and *COASY* represent up to three-quarters of the combined recessive NBIA lifetime risk [11]. Together, these data identify PKAN, CoPAN, PLAN, MPAN, and BPAN as major genetically defined NBIA forms with active therapeutic development. The central question addressed is how genetic and pathway-specific disease mechanisms can guide therapeutic development beyond iron-centered approaches.

Despite this genetic and mechanistic diversity, several neurological features recur across NBIA disorders and remain central to clinical recognition.

Typical clinical manifestations of NBIA vary by genetic subtype, but generally include progressive dystonia, dysarthria, spasticity, parkinsonism, neuropsychiatric abnormalities, cognitive decline, and optic atrophy or retinal degeneration [31]. Onset ranges from infancy to adulthood, and progression may be rapid or slow, sometimes with prolonged periods of relative stability [31].

Cognitive and neuropsychiatric manifestations are clinically relevant across the NBIA spectrum, with intellectual disability, developmental delay, behavioral symptoms, depression, and dementia reported in several subtypes [31,32]. Their recognition is important for patient management and therapeutic development, as cognition, behavior, and adaptive function may represent meaningful clinical outcomes, particularly in early-onset disorders.

NBIA disorders share abnormal iron accumulation in the basal ganglia, mainly in the globus pallidus (GP) and/or substantia nigra (SN), which can be visualized using iron-sensitive Magnetic Resonance Imaging (MRI) sequences [33]. Some forms show characteristic MRI patterns, including the “eye-of-the-tiger” sign in PKAN [34] and T1 hyperintensity with a central hypointense band in the SN and cerebral peduncles in BPAN [35]. Additional abnormalities, including generalized cerebral and cerebellar atrophy, are frequently observed [31]. However, early MRI may be normal in some NBIA forms, as iron accumulation can appear after the first neurological manifestations [31]. Thus, although iron deposition remains central for clinical recognition, its pathogenic role is unresolved and may reflect a convergent downstream mechanism rather than the primary disease trigger [21].

**Figure 1:**
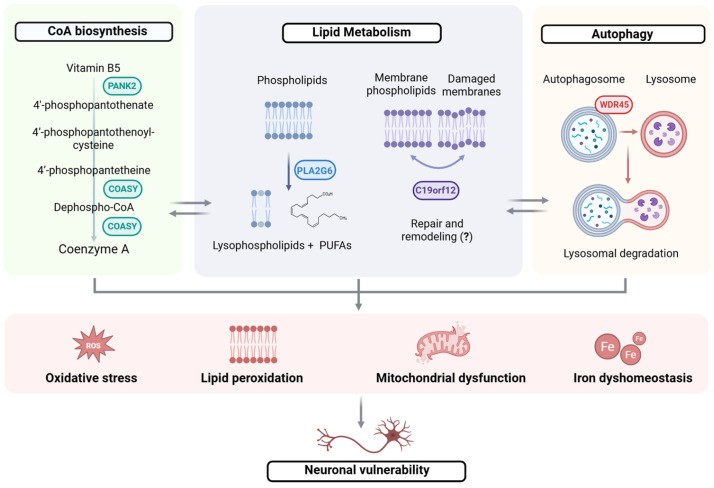
Schematic representation of the major cellular pathways affected in PKAN, CoPAN, PLAN, MPAN, and BPAN. PANK2 and COASY impair CoA biosynthesis, PLA2G6 and C19orf12 affect lipid metabolism and membrane remodeling, and WDR45 disrupts autophagy. These pathway-specific defects converge on oxidative stress, lipid peroxidation, mitochondrial dysfunction, and iron dyshomeostasis, leading to neuronal vulnerability. Created with BioRender (DI Meo, I. (2026) https://BioRender.com/uoobr6s).

Experimental models are increasingly used to define the molecular, cellular, and neurological features of NBIA disorders and to evaluate candidate therapeutic strategies. Here, we discuss how the five major NBIA genes converge on interconnected pathways involving CoA biosynthesis, lipid metabolism, membrane remodeling, mitochondrial dysfunction, autophagy, oxidative stress, lipid peroxidation, and iron dyshomeostasis (Figure 1).

## 3. NBIA Due to Coenzyme A Metabolism Defects

CoA is a central metabolic cofactor required for hundreds of biochemical reactions, including energy production, lipid and fatty acid synthesis, mitochondrial function, and protein acetylation [36]. CoA biosynthesis proceeds through five enzymatic steps (Figure 1). Pantothenate kinase (PANK) catalyzes the first and rate-limiting phosphorylation of pantothenic acid (vitamin B5), generating 4′-phosphopantothenate. Phosphopantothenoylcysteine synthetase (PPCS) then conjugates 4′-phosphopantothenate with cysteine to generate 4′-phosphopantothenoylcysteine, which is decarboxylated by phosphopantothenoylcysteine decarboxylase (PPCDC) to produce 4′-phosphopantetheine. Finally, the bifunctional enzyme CoA synthase (COASY) catalyzes the final two reactions leading to CoA synthesis [37].

Pathogenic variants affecting all enzymes of the CoA biosynthetic pathway have been linked to human diseases and are collectively referred to as inborn errors of CoA biosynthesis [7,38,39]. Among them, *PANK2* and *COASY* variants cause NBIA and may compromise the capacity of cells to sustain CoA-dependent reactions. These include 4′-phosphopantetheinylation of mitochondrial acyl carrier protein (mtACP) and lipoylation of key dehydrogenase complexes, thereby connecting CoA metabolism to mitochondrial dysfunction, altered lipid metabolism, and increased susceptibility to oxidative stress in neurons [40].

### 3.1. PKAN

Pantothenate kinase-associated neurodegeneration (PKAN, OMIM #234002) is among the most prevalent forms of NBIA, with an estimated incidence of approximately 2 per 1,000,000 individuals worldwide [41].

It is caused by biallelic pathogenic variants in the *PANK2* gene on chromosome 20p13 [42], which encodes the pantothenate kinase 2 enzyme and catalyzes the first, rate-limiting reaction in CoA biosynthesis (Figure 1). *PANK2* variants impair CoA metabolism and perturb downstream pathways related to iron/calcium homeostasis, mitochondrial function, lipid metabolism, and reactive oxygen species (ROS) production [43].

Although other PANK isoforms exist (PANK1α, PANK1β, PANK3, PANK4) [44], PANK2 is unique because it localizes to the mitochondrial intermembrane space [45] and responds to mitochondrial acylcarnitines. In particular, palmitoylcarnitine activates PANK2, linking CoA biosynthesis to fatty acid transport into mitochondria [46,47].

PKAN spectrum disorders, including HARP syndrome (hypobetalipoproteinemia, acanthocytosis, retinitis pigmentosa, and pallidal degeneration) [48], are generally classified into classic and atypical forms [6]. The classic form, with onset in the first decade of life, is characterized by rapid progression, loss of ambulation, dystonia, spasticity, and pigmentary retinopathy. It is often associated with severe loss-of-function variants, and earlier onset correlates with more severe cognitive impairment [6,49,50]. The atypical form, with onset usually in the second decade, presents with slower progression. Initial symptoms are often speech impairment and neuropsychiatric manifestations, including obsessive-compulsive symptoms, tics and depression, followed later by motor degeneration. Atypical PKAN is mostly linked to missense variants associated with residual enzymatic activity, although intermediate phenotypes are also recognized [6,49,51].

Diagnosis relies on genetic testing and neuroradiologic examination. Brain MRI in PKAN typically reveals the pathognomonic “eye of the tiger” sign, defined by central pallidal T2 hyperintensity surrounded by T2 hypointensity in the GP, reflecting primary lesions, gliosis, and iron accumulation [34,52]. Computed tomography (CT) scans may also show GP calcifications [53]. Moreover, diffusion tensor imaging (DTI) studies have further linked white matter abnormalities, particularly in the corpus callosum, frontal lobe, medulla oblongata, and pons, to dystonia progression [54].

Different experimental models have been generated to investigate PKAN disease mechanisms. Available models, including yeast, fly, zebrafish, and mouse, reproduced selected molecular or neurological features but failed to fully recapitulate the human phenotype. In zebrafish, morpholino-mediated *Pank2* silencing caused abnormal neuronal development in the telencephalon and diencephalon, along with vascular defects, whereas the CRISPR/Cas9 *Pank2* knock-out (KO) model showed testicular atrophy and anxiety-related behavioral alterations; neither model showed overt neurodegeneration [55,56]. In *Drosophila*, downregulation of the sole *PANK* ortholog, *fumble* (fbl), resulted in severe motor impairment, brain vacuolization, and male sterility, without detectable iron accumulation. This difference may reflect the lack of mitochondrial localization of the fly PANK enzyme [57]. In mice, constitutive *Pank2* loss caused defective spermatogenesis, retinal degeneration in aged animals, reduced weight gain, and mitochondrial ultrastructural abnormalities, as well as perturbation of iron homeostasis and dopamine metabolism in GP-enriched regions, but without any sign of motor deficits or neurodegeneration [58,59]. However, ketogenic diet administration induced PKAN-like motor dysfunction in *Pank2* KO mice, suggesting that metabolic stress can unmask disease-relevant phenotypes [60].

Patient-derived iPSC models have provided complementary insights into human cellular vulnerability. Reprogrammed glutamatergic neurons showed mitochondrial dysfunction but not iron accumulation [61]. In contrast, iron overload was observed in striatal-like medium spiny GABAergic neurons and astrocytes, together with altered mitochondria, oxidative stress, signs of ferroptosis, and impaired endosomal trafficking [62,63]. Interestingly, metabolomics studies in patient plasma further revealed impaired lipid and cholesterol synthesis, as well as elevated plasma lactate and pantothenate, supporting a broader metabolic disturbance in PKAN [64].

### 3.2. CoPAN

COASY protein-associated neurodegeneration (CoPAN, OMIM #615643) is a rare autosomal recessive disorder within the NBIA spectrum. It is caused by biallelic pathogenic variants in *COASY*, located on chromosome 17q21.2 [65]. The gene encodes the bifunctional enzyme 4′-phosphopantetheine adenyltransferase/dephospho-CoA kinase (PPAT/DPCK), or CoA synthase (COASY), which catalyzes the final two steps of CoA biosynthesis in mitochondria, cytoplasm, and nucleus (Figure 1) [7,66]. Biallelic complete loss-of-function variants result in the lethal perinatal condition known as pontocerebellar hypoplasia type 12 (PCH12) [67,68], whereas missense variants retaining residual enzymatic activity cause CoPAN. CoPAN usually manifests in early childhood with movement difficulties and later progresses to bradykinesia, areflexia, dysarthria, and dystonia. Cognitive impairment and additional neuropsychiatric symptoms may also occur [7]. Recent findings have expanded the clinical spectrum to include speech delay, deafness, autism spectrum disorder, neuromuscular involvement, and epilepsy [22].

The “eye of the tiger” sign, classically associated with PKAN, has been reported in some CoPAN patients but is not consistently present. Other MRI findings include GP hypointensity, bilateral hyperintensity, and swelling in the caudate nucleus, putamen, and thalamus. Additional abnormalities may include a smaller corpus callosum, microcephaly, brain atrophy, and frontotemporal and parietal white matter changes [69].

So far, several CoPAN experimental models have been developed. These include *Saccharomyces cerevisiae*, in which PPAT and DPCK enzymatic activities for the synthesis of CoA reside on two different proteins, encoded by *CAB4* and *CAB5* genes, respectively [70]. The deletion of both genes results in a lethal phenotype, consistent with the incompatibility of complete Coasy loss with life [70]. Similarly, the complete absence of *Coasy* in zebrafish is associated with early death due to severe developmental impairment [71]. Together, these findings support an essential role of *COASY* in development.

Since constitutive *Coasy* ablation is embryolethal, conditional KO mouse models have been generated by deleting *Coasy* in neurons or astrocytes using Cre recombinase under cell-type-specific promoters [72,73]. These models recapitulate mitochondrial dysfunction, iron dyshomeostasis, and neurodevelopmental defects, but do not fully capture progressive neurodegeneration [72,73]. To better investigate neurodegenerative processes, a tamoxifen-inducible neuronal KO mouse model was recently generated [74]. This model displayed a more CoPAN-like phenotype, characterized by motor decline, progressive neurodegeneration, brain iron dyshomeostasis, and extensive neuroinflammation, providing an important platform for investigating disease mechanisms and testing therapeutic interventions.

Human iPSC-derived astrocytes have provided complementary disease-relevant evidence. These cells exhibited cytosolic iron accumulation, altered mitochondrial morphology, impaired endosomal trafficking, lipid peroxidation, and cellular senescence [23].

## 4. NBIA Due to Lipid Metabolism Defects

### 4.1. PLAN

PLA2G6-associated neurodegeneration (PLAN) comprises a group of autosomal recessive neurodegenerative disorders caused by biallelic variants in *PLA2G6*, located on chromosome 22, and encoding iPLA2β/iPLA2VI, a calcium-independent phospholipase A2 [8]. PLA2G6 belongs to the phospholipase A2 superfamily and localizes to the cytosol and mitochondria, where it plays a key role in phospholipid metabolism and membrane remodeling (Figure 1) [75]. Phospholipase A2 enzymes hydrolyze acyl-ester bonds of membrane phospholipids, releasing lysophospholipids and free polyunsaturated fatty acids (PUFAs) [76]. Loss of PLA2G6 function impairs membrane phospholipid remodeling and promotes the accumulation of peroxidation-prone lipid species, thereby disrupting neuronal membrane integrity and contributing to neurodegeneration [77,78,79].

Depending on the age of onset and the associated clinical features, PLAN can be classified into infantile neuroaxonal dystrophy (INAD, OMIM #256600), atypical neuroaxonal dystrophy (ANAD, OMIM #610217), and autosomal recessive early-onset Parkinson’s disease PARK14 (EOPD, OMIM #612953) [80].

INAD is usually associated with null alleles and begins early in childhood with rapid motor regression, hypotonia, ataxia, and visual failure, often leading to death within the first decade [34,81]. Neonatal cases have also been reported, presenting congenital hypotonia, severe weakness, and bulbar signs [82]. ANAD is often linked to missense variants, has a slower onset, typically between seven and ten years of age, and is characterized by extrapyramidal signs and intellectual disability [34,83]. By contrast, EOPD usually emerges in adulthood, between 20 and 40 years of age, with neuropsychiatric disturbances, dystonia, and parkinsonism [84,85].

Brain iron accumulation in PLAN is often late, variable, or even absent [86]. Diagnosis therefore relies on integration of clinical presentation, brain MRI, and molecular confirmation. Cerebellar atrophy, axonal spheroids, and the widespread Lewy body pathology represent important neuroradiological and neuropathological hallmarks [34,87].

Established animal models, including *iPLA2β* KO mice and *Drosophila*, show widespread α-synuclein aggregation and motor abnormalities [88,89,90,91,92]. In the mouse model, Pla2g6 deficiency impairs the release of fatty acids from peroxidized lipids, leading to increased oxidative stress and accumulation of cardiolipin-rich inner mitochondrial membrane structures [93].

PLAN patient-derived fibroblasts showed increased oxidative stress and lipid peroxidation. Iron accumulation, accompanied by the presence of lipofuscin granules, has also been observed in induced neurons generated by direct fibroblasts reprogramming [94]. To date, iPSC-based models have been developed only for PLA2G6-associated early-onset Parkinson’s disease. Patient-derived iPSCs and derived dopaminergic neurons exhibited mitochondrial fragmentation, altered bioenergetics, imbalanced calcium homeostasis, and increased susceptibility to apoptosis [24,25,95].

### 4.2. MPAN

Mitochondrial membrane protein-associated neurodegeneration (MPAN, OMIM #624298) is caused by pathogenic variants in *C19orf12*, located on chromosome 19. This disorder is usually inherited as an autosomal recessive trait, although autosomal dominant cases have also been described [10,96].

Clinical manifestations often involve dystonia, spasticity, parkinsonism, motor axonal neuropathy, and retinal and optic abnormalities. In addition to iron accumulation in the GP and SN, neuropathological findings include axonal spheroids, Lewy bodies, and hyperphosphorylated tau in different brain regions [10]. A relatively characteristic MRI feature is a hyperintense band in the GP, corresponding to the internal medullary lamina, which may help distinguish MPAN from other NBIA forms, although rare overlap with PKAN-like “eye-of-the-tiger” patterns has been reported [97].

*C19orf12* encodes a transmembrane protein whose function is still not well clarified [10]. Its subcellular localization to mitochondria, endoplasmic reticulum, and mitochondria-associated membrane (MAM) suggests a role in phospholipid metabolism and membrane remodeling (Figure 1). This hypothesis is supported by high *C19orf12* expression in the adipose tissue, which further increases during adipocyte differentiation. Consistently, *C19orf12* is co-regulated with genes that are primarily involved in fatty acid metabolism [10,98].

Animal models used to study C19orf12 deficiency include *Drosophila* and zebrafish [26]. *Drosophila* has two *C19orf12* orthologs, and only the deletion of both impacts lifespan and climbing performance [99]. Zebrafish has four *C19orf12* orthologs, and the downregulation of the most highly expressed embryonic ortholog causes defective locomotor behavior, altered brain morphology, and decreased survival [100]. Recently, a *C19orf12* KO mouse model has been generated, showing age-dependent axonal spheroids throughout the brain and spinal cord, with accumulation of tangled ER-derived membranes and damaged mitochondria, particularly in dopaminergic neurons. The mice also exhibited brain iron accumulation, α-synuclein accumulation, neuroinflammation, and progressive motor decline starting around six months of life [27].

Cellular models, including MPAN patient fibroblasts and *C19orf12* KO neuroblastoma M17 cells, showed impaired autophagy, altered mitochondrial function, elevated ferrous and ferric iron and ROS levels in the cytosol, and increased vulnerability to ferroptosis [101,102,103]. Recently, patient iPSC-derived midbrain dopaminergic neurons have been generated, revealing iron accumulation, α-synuclein aggregation, axonal swelling, and severe membrane abnormalities. Elevated levels of the major histocompatibility complex class I (MHC-I) were also detected, suggesting a possible contribution of neuronal stress or inflammatory signaling [28].

## 5. NBIA Due to Autophagy Defects

### BPAN

Beta-propeller protein-associated neurodegeneration (BPAN, OMIM #300894) is an X-linked dominant NBIA disorder and is now recognized as one of the most frequently diagnosed forms in recent clinical cohorts [104]. It is caused by pathogenic variants in *WDR45*, located on chromosome X. Most affected individuals are female and carry *de novo* mutations, whereas affected males are usually thought to result from post-zygotic mosaicism or rare surviving hemizygous states [105]. Skewed X-chromosome inactivation may further contribute to the phenotypic variability observed in female patients [105,106].

Typical BPAN MRI findings include T2 hypointensity in the SN and GP, together with T1 hyperintensity of the SN and cerebral peduncles. A characteristic feature is a thin, dark central band surrounded by a halo of brilliance in the SN and the cerebral peduncles. In addition, cerebral and cerebellar atrophy are other frequently observed signs indicating BPAN [104,107].

Formerly referred to as Static Encephalopathy of childhood with Neurodegeneration in Adulthood (SENDA), BPAN is characterized by an onset that typically manifests in early childhood, with global developmental delay, intellectual disability, abnormal behavior, and seizures. Disease progression is usually characterized by a relatively sudden worsening in late adolescence or adulthood, with the emergence of additional neurological symptoms, including progressive dystonia, parkinsonism, and dementia [1].

The gene encodes the WDR45 protein, also known as WIPI4 (WD-repeat domain phosphoinositide interacting protein 4), a member of the autophagy-related (ATG) WIPI proteins involved in autophagosome formation (Figure 1). Because autophagy is involved in synaptic remodeling and plasticity in neurons, disruption of this pathway may contribute to neuronal dysfunction and clinical manifestations in BPAN [108,109].

BPAN animal models include mice, flies, and worms. The cerebral-conditional *Wdr45* KO mouse model showed ubiquitin-positive aggregates, axonal swelling associated with motor neurodegeneration, and learning and memory impairment. Iron accumulation was not detected, probably due to its late manifestation in the disease progression [110]. A recent germline mutant mouse model showed a phenotype similar to that of the conditional model, but also displayed increased endoplasmic reticulum stress and protein aggregation leading to neuronal death [111]. Similarly, deletion of the corresponding *WDR45* orthologs, *Atg18* in yeast and *epg-6* in *C. elegans*, causes defects in autophagy [112,113]. Finally, the *Drosophila* KO model displays impaired locomotor behavior, reduced survival, autophagic dysregulation, and iron dyshomeostasis [29].

Several cellular models have also been used, including human fibroblasts, lymphoblastoid cells, neuroblastoma cell lines, and patient-derived iPSC carrying *WDR45* pathogenic variants. These systems have shown impaired autophagic flux, lysosomal abnormalities, impaired mitochondrial respiration, increased ROS production, altered iron-handling proteins, and iron accumulation [114,115,116,117]. Together, these findings support a mechanistic link between WDR45 dysfunction, autophagy failure, and iron dyshomeostasis.

## 6. From Pathogenesis to Therapy: Emerging Treatment Strategies in Major NBIA Forms

At present, no disease-modifying therapy is available for NBIA disorders, and treatment options remain largely palliative or symptomatic. Supportive care includes physical, occupational, and speech therapy, whereas pharmacological management relies on baclofen for dystonia, levodopa for parkinsonism, botulinum toxin and benzodiazepines for muscle relaxation, and other standard approaches for pain control [118]. Pallidal deep brain stimulation (DBS) has been recognized as a useful treatment for primary and secondary dystonia, with reported benefits in PKAN patients [119].

Magnetic resonance-guided focused ultrasound (MRgFUS) pallidotomy has also shown beneficial outcomes for the treatment of the *status dystonicus* [120]. Nevertheless, both DBS and lesioning procedures remain symptomatic interventions and are generally limited to selected clinical contexts [120].

**Figure 2:**
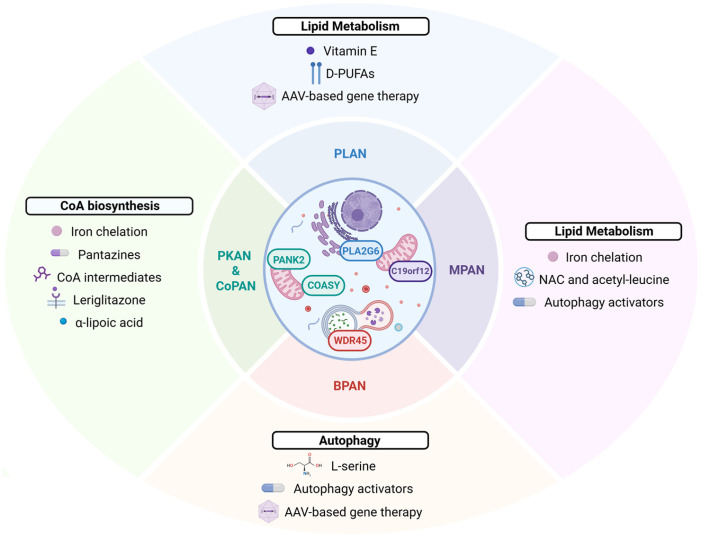
Graphical representation of major NBIA disorders showing the affected genes, protein localization, the associated impaired cellular pathways, and the currently explored therapeutic strategies. Created with BioRender.

Insights from disease models have highlighted potential therapeutic strategies, some of which may be shared across NBIA forms (Figure 2).

Although some of these strategies have shown promising preclinical results, most do not directly address the underlying genetic defects. For this reason, increasing attention is shifting toward genetic and precision-oriented strategies, including gene replacement, gene modulation, and interventions tailored to disease-specific molecular mechanisms. The therapeutic strategies discussed in this section are summarized in Table 2 according to target pathway, evidence level, and translational status.

### 6.1. Symptomatic Management and Iron-Targeting Strategies

Iron plays a vital role in multiple biological processes in the brain, including the synthesis of neurotransmitters, oxidative phosphorylation, and energy production. Nevertheless, excessive iron deposition is an eponymous feature of NBIA disorders and may contribute to oxidative stress, lipid peroxidation, ferroptosis-related vulnerability, and neuronal injury. Iron chelation has therefore been investigated as a potential disease-modifying strategy, particularly in PKAN. However, this approach faces significant hurdles: iron chelators must cross the blood–brain barrier (BBB) and reduce pathological iron excess without causing systemic iron deficiency or regional brain iron depletion [121].

Currently, three iron chelators are clinically available: deferoxamine (DFO), deferasirox (DFS), and deferiprone (DFP), with DFP being the most extensively studied in NBIA because of its ability to cross the BBB.

In a randomized, double-blind, controlled trial involving a large cohort of PKAN patients, 18 months of oral DFP administration significantly reduced GP iron accumulation on MRI, showing a trend towards slower disease progression and maintaining a favorable safety profile (NCT01741532) [122]. The same cohort was subsequently enrolled in an open-label extension study in which all participants received DFP for an additional 18 months. No further differences were observed in patients previously treated with DFP during the initial trial, while patients originally assigned to placebo showed a measurable slowing of disease progression upon initiation of DFP therapy (NCT02174848) [122]. However, clinical efficacy varied considerably among NBIA subtypes. DFP off-label treatment has been reported in two siblings affected by MPAN. Symptom stabilization was observed in only one patient and persisted over four years of follow-up, whereas the treatment was ineffective in the other sibling [123]. This outcome suggests a possible correlation between therapeutic response and individual disease presentation. Similarly, DFP treatment was attempted in two unrelated BPAN patients. One patient showed no remarkable clinical response. For the second patient, presenting milder symptoms, the treatment was interrupted shortly after initiation due to an acute worsening of clinical manifestations [124,125]. Overall, these reports indicate that the clinical utility of DFP remains variable and likely dependent on the underlying genetic and clinical profile.

DFO is characterized by low lipophilicity and high molecular weight, which limit its ability to cross the BBB after systemic parenteral administration [126]. The intranasal administration may bypass the BBB and improve Central Nervous System (CNS) delivery while limiting systemic adverse effects, as demonstrated in AD mouse models [127,128]. However, intranasal DFO requires frequent high-dose administration, causing irritation to the nasal mucosa [126].

DFS has iron-chelating efficacy comparable to that of DFP, but its use is limited by higher dose-related toxicity concerns [126].

Beyond classical chelators, multifunctional compounds with metal-binding, antioxidant, or radical-scavenging properties, including curcumin, capsaicin, S-allylcysteine, or 5-YHEDA, have been explored in other neurodegenerative contexts [126,129,130,131]. However, their relevance to NBIA remains largely speculative because disease-specific preclinical and clinical evidence is lacking.

A complementary strategy is to modulate iron import rather than remove accumulated iron. Transferrin receptor 1 (TfR1) mediates cellular uptake of transferrin-bound iron, and its palmitoylation physiologically limits receptor internalization and iron entry. Impaired TfR1 palmitoylation and recycling were reported in fibroblasts from several NBIA subtypes, suggesting a possible convergent mechanism for pathological iron accumulation [12]. Moreover, PKAN hiPSC-derived astrocytes showed an increase in transferrin uptake associated with altered endocytosis and impaired endosomal trafficking [63]. In this context, pharmacological induction of TfR1 palmitoylation has been proposed as a therapeutic strategy to reduce iron influx. Artesunate restored TfR1 palmitoylation and reduced cellular iron uptake in patient-derived fibroblasts, supporting proof of concept for iron-import modulation [12]. However, this approach remains preclinical, and its translational relevance is uncertain because artemisinin derivatives may also increase oxidative stress, lipid peroxidation, or endoplasmic reticulum stress in vulnerable neuronal contexts [132,133]. Further studies are therefore required before TfR1 palmitoylation can be considered a viable therapeutic target in NBIA.

However, current evidence suggests that iron overload is often a downstream component of NBIA pathogenesis rather than the primary disease driver. Iron-directed therapies may therefore mitigate iron toxicity or slow disease progression, but they are unlikely to provide a definitive treatment when used alone. In the absence of direct genetic correction, more effective strategies will likely require disease-specific or combinatorial approaches able to address the broader metabolic and organelle pathways disrupted in each NBIA subtype.

### 6.2. Metabolic and Pathway-Directed Small-Molecule Strategies

#### 6.2.1. CoA Pathway Restoration in PKAN and CoPAN

Several preclinical studies in PKAN models have explored supplementation with CoA, pantetheine, 4′-phosphopantetheine, or related intermediates to bypass the PANK2-dependent step and restore downstream CoA biosynthesis.

CoA supplementation partially or completely rescued pathological phenotypes in PKAN and, to a lesser extent, CoPAN models, including patient iPSC-derived neurons and astrocytes, *C. elegans*, *Drosophila*, and zebrafish [23,55,61,62,71,134]. However, exogenous CoA is thought to inefficiently cross cell membranes and to be rapidly degraded in serum to 4′-phosphopantetheine [134].

Dietary administration of pantethine, the stable disulfide form of pantothenic acid in zebrafish and *Drosophila* PANK-deficient models, improved the motor phenotype, indicating that partial metabolic rescue can be achieved despite upstream enzymatic deficiency [55,135,136]. In addition, pantethine supplementation via drinking water prevented motor neurodegeneration and significantly ameliorated the phenotype in *Pank2* KO mice subjected to a ketogenic diet-induced metabolic stress [60].

Studies on *Drosophila* and *Pank2* KO mice showed that oral administration of the membrane-permeable CoA intermediate 4′-phosphopantetheine corrected the primary CoA pathway-related biomarkers and ameliorated phenotypical or molecular alterations, as well as rescued secondary perturbations in iron homeostasis, dopamine metabolism, and mitochondrial function [134,137]. Similarly, acetyl-4′-phosphopantetheine was able to prevent or reverse the PKAN-related phenotype in flies and mice [138]. Importantly, precursor-based strategies may be less straightforward in CoPAN than in PKAN, because COASY catalyzes the final two steps of CoA biosynthesis and may still be required to convert downstream intermediates into CoA.

Based on these encouraging preclinical findings, a randomized, double-blind, placebo-controlled clinical trial evaluated oral fosmetpantotenate in a large cohort of PKAN patients. Fosmetpantotenate was designed to replace phosphopantothenate, thereby bypassing the defective enzymatic step. Although the treatment was safe and well tolerated, it failed to demonstrate clinical efficacy, showing no significant improvement in symptoms or slowing of disease progression [139]. More recently, an ongoing phase 2 clinical trial (NCT04182763) has been designed primarily to assess safety, tolerability, and pharmacodynamic biomarkers in PKAN patients treated with 4′-phosphopantetheine [140].

A complementary strategy is PANK activation, which aims to increase CoA biosynthesis by stimulating residual or alternative pantothenate kinase isoforms rather than supplying downstream intermediates. Pantazines are small-molecule PANK activators designed to stabilize catalytically active enzyme conformations and overcome feedback inhibition by acetyl-CoA [141,142,143]. This approach is particularly attractive for PKAN because PANK1 and PANK3 remain genetically intact, but its clinical utility will depend on brain exposure, long-term safety, and the ability to restore CoA-dependent pathways in the most vulnerable neuronal populations.

#### 6.2.2. PPARγ Activation and Mitochondrial Support in PKAN and CoPAN

Leriglitazone is a brain-penetrant peroxisome proliferator-activated receptor gamma (PPARγ) agonist that has been investigated as a mitochondrial and anti-inflammatory strategy in CoA-related NBIA disorders [144]. In PKAN patient-derived astrocytes, leriglitazone restored mitochondrial respiration, reduced cytosolic iron overload, and increased cell viability [145]. These effects suggested that PPARγ activation may counteract downstream consequences of impaired CoA metabolism, including mitochondrial dysfunction and iron dyshomeostasis. The therapeutic potential of leriglitazone was also evaluated in an inducible neuronal *Coasy* KO mouse model of CoPAN. In this model, leriglitazone improved motor performance, restored iron homeostasis, and mitigated neurodegeneration and neuroinflammation. However, treatment did not extend survival, indicating that modulation of downstream pathways was insufficient to fully reverse the disease phenotype caused by the complete absence of the protein [74]. This discrepancy supports the view that PPARγ activation may provide partial disease modification but is unlikely to replace strategies directly targeting the primary genetic defect.

#### 6.2.3. Autophagy Modulation in BPAN and MPAN

For BPAN, autophagy modulation represents a direct therapeutic rationale because WDR45/WIPI4 directly participates in autophagosome formation and autophagy-related membrane dynamics [112]. In *WDR45*-deficient cellular and mouse models, mTOR inhibition with rapamycin reduced endoplasmic reticulum stress and alleviated neuronal death [111].

Among repurposable metabolic interventions, L-serine has emerged as a candidate molecule due to its role in CNS development, synaptic plasticity, and neuronal signaling [146,147]. This amino acid selectively activates autophagic–lysosomal enzymes such as cathepsins B and L, thereby increasing cellular proteolytic efficiency [148]. It has been associated with antioxidant and neuroprotective properties in vitro, in vivo, and in clinical trials for neurodegenerative disorders, including Amyotrophic Lateral Sclerosis (ALS) and Hereditary Sensory and Autonomic Neuropathy type 1 (HSAN1) [149,150]. In BPAN patient fibroblasts, L-serine rescued lysosomal dysfunction by reducing lysosomal enlargement, restoring lysosomal enzymatic activity, mitigating oxidative stress, and significantly decreasing iron and oxidized lipid accumulation, including pathological lipofuscin aggregates derived from lipid peroxidation [151].

Autophagy-modulating approaches may also be relevant to MPAN, although *C19orf12* is not a canonical autophagy gene. Patient-derived MPAN fibroblasts showed impaired autophagy initiation, which was corrected by carbamazepine and other autophagy-modulating compounds [103].

Collectively, these findings indicate that autophagy modulation represents a rational therapeutic strategy for BPAN and a potentially actionable downstream target in MPAN.

### 6.3. Antioxidant and Anti-Ferroptotic Strategies

#### 6.3.1. α-Lipoic Acid as an Antioxidant Strategy in PKAN

α-lipoic acid (ALA) is an antioxidant compound that reduces pro-inflammatory signaling and ROS production, thereby enhancing cellular survival and mitochondrial function. Given its neuroprotective properties, ALA has been evaluated in fibroblasts and induced neurons derived from PKAN patients. In these models, ALA treatment reduced iron accumulation and lipid peroxidation, improved mitochondrial function, and increased residual *PANK2* expression [152]. Consistently, ALA administration significantly reduced iron levels in a zebrafish model of induced iron accumulation [153]. These results suggest that ALA may deserve further evaluation, particularly in PKAN patients retaining residual protein activity.

#### 6.3.2. D-PUFAs and Vitamin E for Lipid-Peroxidation Control in PLAN

Autoxidation of PUFAs produces toxic lipid peroxidation products that can damage cellular and mitochondrial membranes. This mechanism is particularly relevant to PLAN because PLA2G6 deficiency impairs phospholipid remodeling and increases vulnerability to oxidative membrane damage. Deuterated PUFAs (D-PUFAs), in which allylic hydrogens have been replaced by deuterium, were developed to slow lipid peroxidation and stabilize membrane lipids [154].

Within the NBIA spectrum, deuterated linoleic acid rescued locomotor abnormalities in the *Drosophila* PLAN model. Beneficial effects were also observed in PLAN patient fibroblasts, where treatment reduced lipid peroxidation and improved mitochondrial membrane potential [88,121,155]. Oral administration of deuterated linoleic acid was reported in two INAD subjects, with preliminary evidence of clinical stabilization [156].

A combination of antioxidant molecules, including vitamin E and omega-3, was reported to reduce oxidative stress and lipid peroxidation in PLAN patient fibroblasts and induced neurons generated by direct reprogramming. Vitamin E also partially reversed senescent morphology, prevented iron and lipofuscin accumulation, and reduced mitochondrial network fragmentation [94].

Similar outcomes resulted from the treatment of PKAN and BPAN cultured fibroblasts with the same antioxidants, and clinical stabilization has also been described in patients receiving these supplements in combination with baseline neurological medication [157,158,159]. Although these findings support a common rationale across different NBIA forms, antioxidant supplementation should be considered a supportive or pathway-modulating strategy rather than a corrective treatment for the primary genetic defect.

#### 6.3.3. NAC and Acetyl-Leucine as Exploratory Strategies in MPAN

N-acetyl-L-cysteine (NAC) is a thiol antioxidant that directly scavenges reactive oxygen species and serves as a precursor for cysteine and glutathione synthesis [160]. In *C19orf12* KO M17 neuroblastoma cells, NAC supplementation reduced lipid peroxidation and partially rescued mitochondrial structural and respiratory abnormalities [101]. These findings support oxidative stress and ferroptosis-related damage as actionable downstream targets in MPAN, although the evidence remains limited to cellular models.

Acetyl-leucine has recently emerged as an exploratory candidate in MPAN based on patient-derived neuronal models. In midbrain dopaminergic neurons generated from MPAN patient iPSCs, treatment with acetyl-leucine reduced MHC-I levels, suggesting a possible effect on neuronal stress or neuroinflammatory signaling [28]. However, this finding remains preliminary, and it is not yet clear whether MHC-I reduction reflects a disease-modifying mechanism, a secondary stress response, or a model-specific readout.

### 6.4. Gene-Based and Precision Genetic Strategies

Gene-based therapies represent an attractive therapeutic approach for monogenic neurological conditions, particularly for recessive disorders [161]. In NBIA, these strategies could correct the primary molecular defect rather than merely modulate downstream consequences such as iron dyshomeostasis, oxidative stress, mitochondrial dysfunction, or impaired autophagy. However, their translation remains challenging because effective treatment will require early diagnosis, adequate central nervous system delivery, appropriate cell-type targeting, durable expression, and careful control of transgene dosage.

The four fundamental approaches to gene therapy, namely gene silencing, gene replacement, gene addition, and gene editing, are based on the delivery of genetic material into the cells to replace defective genes, introduce new genes, or modify the expression of existing genes through the use of viral or non-viral vectors [162,163,164]. Among these strategies, adeno-associated virus (AAV)-mediated gene replacement currently represents the most advanced approach for recessive NBIA forms, whereas allele-selective silencing, antisense oligonucleotides, and genome editing remain less mature or applicable only to specific genetic contexts.

#### AAV-Mediated Gene Replacement

Gene therapy has achieved the most advanced preclinical progress primarily in PLAN and BPAN. These approaches use AAV vectors to deliver functional copies of the defective genes, aiming to restore cellular homeostasis in affected neuronal populations.

A landmark study in PLAN used AAV-mediated delivery of human *PLA2G6* cDNA to rescue the phenotype of a KO mouse model. The therapeutic transgene was driven by the ubiquitous EF1α promoter and packaged into the synthetic AAV-PHP.eB serotype, known for its robust BBB penetration in mice. Intracerebroventricular (ICV) administration in pre-symptomatic pups improved sensorimotor function, extended survival, and preserved cerebellar Purkinje cells, which are key sites of pathology due to phospholipid metabolism defects [165]. However, it has also been demonstrated that the PhP.eB serotype showed very high transduction efficacy in selected mouse strains but poor brain transduction in non-human primates, limiting its direct clinical translation and supporting the need for clinically relevant AAV capsids and delivery routes [166].

In parallel with academic preclinical studies, an investigational AAV9-based *PLA2G6* gene therapy program for INAD has entered translational development. The program is designed for one-time cerebrospinal fluid administration and has received Orphan Drug Designation from both the U.S. Food and Drug Administration (https://www.accessdata.fda.gov/scripts/opdlisting/oopd/detailedIndex.cfm?cfgridkey=960923, accessed on 7 October 2026) and the European Commission (https://ec.europa.eu/health/documents/community-register/html/o2845.htm, accessed on 7 October 2026). However, peer-reviewed clinical efficacy data are not yet available, and this program should therefore be regarded as a translational pipeline candidate rather than an established therapeutic approach.

Similarly, gene therapy for BPAN has yielded encouraging preclinical data. Carisi et al. developed a novel knock-in mouse model harboring the WDR45 c830 + 1g > a hemizygous variant, which recapitulates early-onset hyperactivity, as shown by open-field testing, and autophagy blockade, as indicated by reduced LC3-II and p62 accumulation. Pre-symptomatic ICV delivery of AAV9-*WDR45* under the synthetic and constitutive JeT promoter was able to prevent the neurological alteration and correct the autophagic markers without overt adverse effects, providing preclinical proof-of-concept for *WDR45* gene replacement in BPAN [167].

Gene replacement strategies for NBIA caused by inborn errors of CoA biosynthesis remain at an earlier stage of development. AAV9-mediated delivery of full-length human *PANK2* has been proposed as a rational strategy for PKAN, and early preclinical work with stereotactic delivery to the GP of the *Pank2* KO mouse model has been announced [121]. However, peer-reviewed efficacy and safety data have not yet been published.

Similarly, no published studies have yet tested AAV-based *COASY* gene replacement in disease-relevant CoPAN models. However, available in vitro and in vivo complementation studies provide initial support for the feasibility of this approach. Human *COASY* expression restored metabolic defects in mutant yeast models, as well as rescued developmental, mitochondrial, and motor abnormalities in zebrafish and *Drosophila Coasy*/*Dpck*-deficient models [30,70,71]. These findings provide cross-species evidence that restoration of COASY activity can correct key consequences of enzyme deficiency, thereby providing a foundation for future preclinical validation.

## 7. Translational Gaps and Future Perspective

As our understanding of NBIA pathogenesis improves, therapeutic development is progressively moving from symptomatic management toward mechanism-based interventions. However, this transition remains incomplete. Iron chelation, metabolic supplementation, antioxidant strategies, autophagy modulation, and gene replacement approaches have all provided useful proof-of-concept data, but none has yet established broad clinical disease modification across NBIA disorders [122,140,151,156,165]. A central challenge is that most available treatments target downstream consequences of disease, whereas neuronal dysfunction may already be advanced by the time patients receive a diagnosis.

Therapeutic timing is likely to be one of the strongest determinants of efficacy. Several NBIA forms begin before iron accumulation becomes radiologically evident, and irreversible neuronal loss may precede overt clinical deterioration. This is particularly relevant for early-onset disorders, in which motor decline, developmental impairment, or neurodegeneration can progress rapidly. Therefore, future therapies will require earlier molecular diagnosis, better genotype-based risk stratification, and disease-stage-specific treatment strategies. Interventions that are ineffective in advanced disease may still be valuable if administered before extensive neuronal loss has occurred [168].

A second major limitation is the lack of robust translational biomarkers. Brain MRI remains central for diagnosis and for monitoring iron accumulation, but radiological improvement does not necessarily predict clinical benefit. This mismatch was evident in iron-chelation studies, where reduced pallidal iron did not consistently translate into substantial functional improvement. Future efforts should prioritize the identification of simple, reliable biomarkers that more directly reflect disease-relevant biology, including quantitative MRI, plasma and/or Cerebral Spinal Fluid (CSF) circulating biomarkers, and omics-based signatures. Such biomarkers are needed not only for efficacy assessment but also for dose selection, patient stratification, and the identification of outcome measures for future clinical studies [169,170].

Natural history studies have been published for PKAN, BPAN, and PLAN, providing an essential framework for patient stratification, outcome selection, and trial design [171,172,173,174]. However, available data remain limited by small cohorts, phenotypic heterogeneity, and variable progression rates. Future studies should further harmonize longitudinal clinical scales, MRI protocols, digital motor measures, caregiver-reported outcomes, and biomarker-supported endpoints to improve the sensitivity of trials in ultra-rare NBIA disorders. Recent Quantitative Susceptibility Mapping (QSM) data in genetically confirmed NBIA patients further support the potential value of quantitative MRI for measuring basal ganglia iron deposition and developing imaging biomarkers for natural history studies and future trials [175].

Given the high global burden of mental disorders and their major contribution to disability, neuropsychiatric and cognitive outcomes should be systematically captured in NBIA natural history studies and interventional trials [176]. This is particularly important for disorders such as BPAN, PKAN, CoPAN, PLAN, and MPAN, in which cognitive decline, developmental impairment, behavioral symptoms, or psychiatric manifestations may contribute substantially to disease burden.

Preclinical development faces parallel limitations. Many cellular and animal models reproduce selected disease mechanisms but fail to capture the full human phenotype. PKAN mouse models, for example, have provided valuable biochemical information but only limited neurodegenerative features [58]. Conversely, severe developmental models may be useful for pathway validation but less suitable for testing post-symptomatic therapeutic rescue. Disease-relevant human iPSC-derived cells and animal models should therefore be integrated rather than used in isolation. For gene-based therapies, preclinical models must also define CNS biodistribution, target-cell requirements, therapeutic expression windows, immunogenicity, and long-term safety.

Gene replacement is among the most rational strategies for recessive NBIA disorders, but its translation will not be straightforward. AAV-based approaches for PLAN and BPAN have provided encouraging preclinical evidence, whereas PKAN and CoPAN remain at earlier stages [165,167]. For these disorders, the key unresolved issues are not only vector delivery, dosage control, timing of intervention, and durability of expression, but also the high development and manufacturing costs associated with ultra-rare diseases. Emerging platforms, including mRNA delivery through lipid nanoparticles (LNPs), may eventually provide alternative strategies with potential advantages in affordability, safety, and reversibility [177].

Epigenetic reactivation and gene-modifying approaches are even less mature. In BPAN, reactivation of the silent wild-type *WDR45* allele represents an interesting concept because of X-linked disease biology, but current evidence remains cellular and far from clinical implementation [178]. Moreover, genome-editing approaches or gene expression modulation by antisense oligonucleotides (ASOs) are currently more speculative and should be viewed as long-term perspectives rather than near-term therapeutic options [126,179].

Given the mechanistic complexity of NBIA, single interventions may not be sufficient. The most plausible future strategy is combination therapy, in which disease-specific correction is paired with modulation of convergent downstream pathways. For example, gene replacement or CoA-pathway restoration may need to be combined with approaches targeting mitochondrial dysfunction, lipid peroxidation, iron dyshomeostasis, ferroptosis, autophagy defects, or neuroinflammation [180].

Together, these gaps define the main priorities for the next phase of NBIA translational research. The field now needs models that predict human therapeutic response, biomarkers that capture disease activity, and trials designed around early intervention and small patient populations. Only by integrating causal therapies with pathway-modulating strategies will it be possible to move beyond symptomatic treatment toward clinically significant disease modification.

## 8. Conclusions

NBIA disorders illustrate how a shared neuroradiological feature can arise from different genetic and molecular defects. Although brain iron accumulation remains central to diagnosis, current evidence indicates that iron dyshomeostasis is often part of a broader network of downstream pathogenic events involving CoA metabolism, lipid remodeling, mitochondrial dysfunction, autophagy impairment, vesicle trafficking defects, oxidative stress, and ferroptosis-related vulnerability. This concept has important therapeutic implications because strategies focused exclusively on iron removal are unlikely to provide broad disease modification across genetically distinct NBIA forms.

Therapeutic development is therefore moving toward mechanism-based approaches, including CoA-pathway restoration, mitochondrial and antioxidant support, lipid-peroxidation control, autophagy modulation, and gene replacement. These strategies have generated promising preclinical results, but clinical translation remains limited by disease rarity, phenotypic heterogeneity, incomplete disease models, lack of robust biomarkers, and the difficulty of treating patients before irreversible neuronal damage occurs. Future progress will require earlier molecular diagnosis, disease-stage-specific intervention, harmonized natural history data, and trial designs adapted to ultra-rare populations. Finally, effective treatment will likely depend on combining causal or pathway-restoring therapies with interventions targeting convergent downstream mechanisms.

## Figures and Tables

**Table 1:** Molecular classification of NBIA disorders.

Disease	Gene	Inheritance	Protein Function	Reference
**Iron homeostasis**				
Aceruloplasminaemia	*CP*	AR	Iron oxidation	[3]
Neuroferritinopathy (NF)	*FTL*	AD	Cellular iron storage	[4]
NBIA9	*FTH1*	AD	Ferroxidase activity	[5]
**CoA biosynthesis**				
Pantothenate kinase-associated neurodegeneration (PKAN)	*PANK2*	AR	Coenzyme A biosynthesis	[6]
COASY protein-associated neurodegeneration (CoPAN)	*COASY*	AR	Coenzyme A biosynthesis	[7]
**Lipid metabolism**				
PLA2G6-associated neurodegeneration (PLAN)	*PLA2G6*	AR	Phospholipid remodeling	[8]
Fatty acid hydroxylase-associated neurodegeneration (FAHN)	*FA2H*	AR	Hydroxylation of fatty acids, myelin formation	[9]
Mitochondrial membrane protein-associated neurodegeneration (MPAN)	*C19orf12*	AR	Lipid metabolism	[10]
Leukoencephalopathy with dystonia and motor neuropathy	*SCP2*	AR	Breakdown of branched-chain fatty acids	[11]
NBIA8	*CRAT*	AR	Carnitine acetyltransferase	[12]
SLC27A3 deficiency	*SLC27A3*	AR	Fatty acid transport	[13]
Mitochondrial Enoyl-CoA Reductase Protein-Associated Neurodegeneration (MEPAN)	*MECR*	AR	Mitochondrial fatty acid synthesis	[14]
**Autophagy**				
β-propeller-associated neurodegeneration (BPAN)	*WDR45*	XL	Autophagosome formation	[15]
Kufor–Rakeb disease (KRS)	*ATP13A2*	AR	Lysosomal cation pump, autophagosome formation	[16]
Spastic paraplegia 50 (SPG-50)	*AP4M1*	AR	Vesicle formation	[17]
NBIA7	*REPS1*	AR	Endocytosis and vesicle transport	[18]
**Other**				
Woodhouse–Sakati syndrome (WSS)	*DCAF17*	AR	Protein ubiquitination	[19]
Jaberi–Elahi syndrome	*GTPBP2*	AR	Not defined	[20]

AR: autosomal recessive, AD: autosomal dominant, XL: X-linked.

**Table 2:** Therapeutic strategies for major NBIA disorders: targets, evidence level, and translational status.

Therapeutic Strategy	Main Target/Pathway	NBIA Form(s)	Evidence Level	Translational Status
Iron chelation	Iron dyshomeostasis	Mainly PKAN; limited reports in MPAN/BPAN	Human studies/case reports	Variable clinical benefit
CoA pathway restoration	CoA biosynthesis	PKAN, CoPAN	Preclinical data in CoPAN and PKAN; clinical trial in PKAN	Clinical trial
PANK activation	CoA biosynthesis	PKAN	Preclinical	Early translational development
PPARγ activation/mitochondrial support	Mitochondrial dysfunction, inflammation, iron handling	PKAN, CoPAN	Preclinical	-
Autophagy modulation	Autophagy-lysosomal dysfunction	BPAN, MPAN	Preclinical	-
Antioxidant and anti-ferroptotic strategies	Lipid peroxidation, oxidative stress, ferroptosis	PKAN, PLAN, BPAN, MPAN	Preclinical, limited patient reports	Supportive/preclinical
AAV-mediated gene replacement	Primary genetic defect	PLAN, BPAN; conceptual for PKAN/CoPAN	Advanced preclinical for PLAN/BPAN	Translational development

## Data Availability

No new data were created or analyzed in this study. Data sharing is not applicable to this article.

## Abbreviations

The following abbreviations are used in this manuscript:

NBIA	Neurodegeneration with brain iron accumulation
CP	Ceruloplasmin
FTL	Ferritin light chain
FTH1	Ferritin heavy chain
PKAN	Pantothenate kinase-associated neurodegeneration
CoPAN	COASY protein-associated neurodegeneration
BPAN	β-propeller-associated neurodegeneration
MPAN	mitochondrial membrane protein-associated neurodegeneration
PLAN	PLA2G6-associated neurodegeneration
INAD	Infantile neuroaxonal dystrophy
ANAD	Atypical neuroaxonal dystrophy
EOPD	Early-onset Parkinson’s disease
MECR	Mitochondrial trans-2-enoyl-CoA reductase
GP	Globus pallidus
SN	Substantia nigra
PPCS	Phosphopantothenoylcysteine synthetase
PPCDC	Phosphopantothenoylcysteine decarboxylase
PPAT	4′-phosphopantetheine adenyltransferase
DPCK	Dephospho-CoA kinase
PUFA	Polyunsaturated fatty acids
MAM	Mitochondria-associated membrane
DFO	Deferoxamine
DFS	Deferasirox
DFP	Deferiprone
TfR1	Transferrin receptor 1
PPARγ	Peroxisome proliferator-activated receptor gamma
ALA	α -lipoic acid
NAC	N-acetyl-L-cysteine
AAV	Adeno-associated virus
HSAN1	Hereditary sensory and autonomic neuropathy type 1
ASOs	Antisense oligonucleotides

## References

[1] Hayflick S.J., Kurian M.A., Hogarth P. (2018). Neurodegeneration with Brain Iron Accumulation. Handb. Clin. Neurol..

[2] Arber C.E., Li A., Houlden H., Wray S. (2016). Review: Insights into Molecular Mechanisms of Disease in Neurodegeneration with Brain Iron Accumulation: Unifying Theories. Neuropathol. Appl. Neurobiol..

[3] Gitlin J.D. (1998). Aceruloplasminemia. Pediatr. Res..

[4] Burn J., Chinnery P.F. (2006). Neuroferritinopathy. Semin. Pediatr. Neurol..

[5] Shieh J.T., Tintos-Hernandez J.A., Murali C.N., Penon-Portmann M., Flores-Mendez M., Santana A., Bulos J.A., Du K., Dupuis L., Damseh N. (2023). Heterozygous Nonsense Variants in the Ferritin Heavy-Chain Gene FTH1 Cause a Neuroferritinopathy. HGG Adv..

[6] Hayflick S.J. (2003). Unraveling the Hallervorden-Spatz Syndrome: Pantothenate Kinase-Associated Neurodegeneration Is the Name…. Curr. Opin. Pediatr..

[7] Dusi S., Valletta L., Haack T.B., Tsuchiya Y., Venco P., Pasqualato S., Goffrini P., Tigano M., Demchenko N., Wieland T. (2014). Exome Sequence Reveals Mutations in CoA Synthase as a Cause of Neurodegeneration with Brain Iron Accumulation. Am. J. Hum. Genet..

[8] Morgan N.V., Westaway S.K., Morton J.E.V., Gregory A., Gissen P., Sonek S., Cangul H., Coryell J., Canham N., Nardocci N. (2006). PLA2G6, Encoding a Phospholipase A2, Is Mutated in Neurodegenerative Disorders with High Brain Iron. Nat. Genet..

[9] Kruer M.C., Paisán-Ruiz C., Boddaert N., Yoon M.Y., Hama H., Gregory A., Malandrini A., Woltjer R.L., Munnich A., Gobin S. (2010). Defective *FA2H* Leads to a Novel Form of Neurodegeneration with Brain Iron Accumulation (NBIA). Ann. Neurol..

[10] Hartig M.B., Iuso A., Haack T., Kmiec T., Jurkiewicz E., Heim K., Roeber S., Tarabin V., Dusi S., Krajewska-Walasek M. (2011). Absence of an Orphan Mitochondrial Protein, C19orf12, Causes a Distinct Clinical Subtype of Neurodegeneration with Brain Iron Accumulation. Am. J. Hum. Genet..

[11] Kolarova H., Tan J., Strom T.M., Meitinger T., Wagner M., Klopstock T. (2022). Lifetime Risk of Autosomal Recessive Neurodegeneration with Brain Iron Accumulation (NBIA) Disorders Calculated from Genetic Databases. eBioMedicine.

[12] Drecourt A., Babdor J., Dussiot M., Petit F., Goudin N., Garfa-Traoré M., Habarou F., Bole-Feysot C., Nitschké P., Ottolenghi C. (2018). Impaired Transferrin Receptor Palmitoylation and Recycling in Neurodegeneration with Brain Iron Accumulation. Am. J. Hum. Genet..

[13] Travaglini L., Jeon C., Rizza T., Novelli A., Specchio N., Piluso A., Bertini E., Iuso A., Garone G. (2025). Biallelic Variants in SLC27A3 Cause a Complex Form of Neurodegeneration with Brain Iron Accumulation. Mov. Disord..

[14] Heimer G., Kerätär J.M., Riley L.G., Balasubramaniam S., Eyal E., Pietikäinen L.P., Hiltunen J.K., Marek-Yagel D., Hamada J., Gregory A. (2016). MECR Mutations Cause Childhood-Onset Dystonia and Optic Atrophy, a Mitochondrial Fatty Acid Synthesis Disorder. Am. J. Hum. Genet..

[15] Haack T.B., Hogarth P., Gregory A., Prokisch H., Hayflick S.J. (2013). BPAN. International Review of Neurobiology.

[16] Schneider S.A., Paisan-Ruiz C., Quinn N.P., Lees A.J., Houlden H., Hardy J., Bhatia K.P. (2010). *ATP13A2* Mutations (PARK9) Cause Neurodegeneration with Brain Iron Accumulation. Mov. Disord..

[17] Roubertie A., Hieu N., Roux C.-J., Leboucq N., Manes G., Charif M., Echenne B., Goizet C., Guissart C., Meyer P. (2018). AP4 Deficiency: A Novel Form of Neurodegeneration with Brain Iron Accumulation?. Neurol. Genet..

[18] Khan H., Ilyas M., Qasim H., Zeb H., Israr M., Iqbal A., Ullah A., Ullah A., Dominik N., Houlden H. (2025). Whole Exome Sequencing Identifies a Novel Variant Causing Neurodegeneration with Brain Iron Accumulation Syndrome (NBIA) in a Consanguineous Pashtun Family. Neurogenetics.

[19] Messina C. (2025). Woodhouse-Sakati Syndrome: A Review. Rev. Neurol..

[20] Manoochehri J., Shiri A., Khoddam S., Aghasipour M., Kamal N., Jafari Khamirani H., Dastgheib S.A., Dianatpour M., Tabei S.M.B. (2024). Jaberi-Elahi Syndrome: Exploring a Novel GTPBP2 Mutation and a Literature Review. Eur. J. Med. Genet..

[21] Levi S., Tiranti V. (2019). Neurodegeneration with Brain Iron Accumulation Disorders: Valuable Models Aimed at Understanding the Pathogenesis of Iron Deposition. Pharmaceuticals.

[22] Cavestro C., Morra F., Legati A., D’Amato M., Nasca A., Iuso A., Lubarr N., Morrison J.L., Wheeler P.G., Serra-Juhé C. (2024). Emerging Variants, Unique Phenotypes, and Transcriptomic Signatures: An Integrated Study of *COASY*-associated Diseases. Ann. Clin. Transl. Neurol..

[23] Cozzi A., Santambrogio P., Moro A.S., Pelagatti A., Rubio A., Balestrucci C., Di Meo I., Tiranti V., Levi S. (2025). Fibroblasts and hiPS-Derived Astrocytes from CoPAN Patients Showed Different Levels of Iron Overload Correlated with Senescent Phenotype. Glia.

[24] Lin Z.-H., Xue N.-J., Liu Y., Zhang F., Si X.-L., Zheng R., Gu L.-Y., Li Y.-L., Fan Y., Tian J. (2026). Parkinson’s Disease-Associated PLA2G6 Protects IP3R1 Protein to Control ER-Mitochondria Tethering and Ca^2+^ Transfer. Nat. Commun..

[25] Musthafa T., Nizami S.K., Mishra A., Hasan G., Gopurappilly R. (2025). Altered Mitochondrial Bioenergetics and Calcium Kinetics in Young-Onset PLA2G6 Parkinson’s Disease iPSCs. J. Neurochem..

[26] Gnutti B., Iuso A., Angelini C., Finazzi D. (2025). An Update and Perspectives on Mitochondrial Membrane Protein-Associated Neurodegeneration and C19orf12 Research. Brain Sci..

[27] Wu F., Siedlak S.L., Bhatta S., Khaled S., Shao C., Torres S., Fujioka H., Wang W. (2025). Loss of Mouse C19orf12 Homolog Disturbs Tubular ER Homeostasis and Leads to Neuroaxonal Dystrophy. Acta Neuropathol. Commun..

[28] Heger L.M., Kertess L., Kaufhold C., Gubinelli F., Cardona-Alberich A., Özata G., Müller S.A., Tschirner S.K., Stehling O., Schifferer M. (2025). Patient-Derived Neurons Exhibit α-Synuclein Pathology and Previously Unrecognized Major Histocompatibility Complex Class I Elevation in Mitochondrial Membrane Protein–Associated Neurodegeneration. Mov. Disord..

[29] Celle M., Aniorte S., Issa A.-R., Falabregue M., Jin H., Sanchez-Mirasierra I., Ding S., Soukup S.-F., Seugnet L., Liao L. (2026). A Dwdr45 Knock-out Drosophila Model to Decipher the Role of Autophagy in BPAN. Hum. Mol. Genet..

[30] Shao Y., Hu J., Yan K., Zheng K., Sha W., Wang J., Wu J., Huang Y. (2025). Impaired Mitochondrial Integrity and Compromised Energy Production Underscore the Mechanism Underlying CoASY Protein-Associated Neurodegeneration. Cell. Mol. Life Sci..

[31] Gregory A., Kurian M.A., Wilson J., Hayflick S., Adam M.P., Bick S., Mirzaa G.M., Pagon R.A., Wallace S.E., Amemiya A. (1993). Neurodegeneration with Brain Iron Accumulation Disorders Overview. GeneReviews^®^.

[32] Tonekaboni S.H., Mollamohammadi M. (2014). Neurodegeneration with brain iron accumulation: An overview. Iran. J. Child. Neurol..

[33] Lee J.-H., Yun J.Y., Gregory A., Hogarth P., Hayflick S.J. (2020). Brain MRI Pattern Recognition in Neurodegeneration with Brain Iron Accumulation. Front. Neurol..

[34] Gregory A., Polster B.J., Hayflick S.J. (2009). Clinical and Genetic Delineation of Neurodegeneration with Brain Iron Accumulation. J. Med. Genet..

[35] Kimura Y., Sato N., Sugai K., Maruyama S., Ota M., Kamiya K., Ito K., Nakata Y., Sasaki M., Sugimoto H. (2013). MRI, MR Spectroscopy, and Diffusion Tensor Imaging Findings in Patient with Static Encephalopathy of Childhood with Neurodegeneration in Adulthood (SENDA). Brain Dev..

[36] Czumaj A., Szrok-Jurga S., Hebanowska A., Turyn J., Swierczynski J., Sledzinski T., Stelmanska E. (2020). The Pathophysiological Role of CoA. Int. J. Mol. Sci..

[37] Strauss E. (2010). Coenzyme A Biosynthesis and Enzymology. Comprehensive Natural Products II: Chemistry and Biology.

[38] Bravo-Alonso I., Morin M., Arribas-Carreira L., Álvarez M., Pedrón-Giner C., Soletto L., Santolaria C., Ramón-Maiques S., Ugarte M., Rodríguez-Pombo P. (2023). Pathogenic Variants of the Coenzyme A Biosynthesis-Associated Enzyme Phosphopantothenoylcysteine Decarboxylase Cause Autosomal-Recessive Dilated Cardiomyopathy. J. Inherit. Metab. Dis..

[39] Lok A., Fernandez-Garcia M.A., Taylor R.W., French C., MacFarland R., Bodi I., Champion M., Josifova D., Raymond F.L., Iuso A. (2022). Novel Phosphopantothenoylcysteine Synthetase (PPCS) Mutations with Prominent Neuromuscular Features: Expanding the Phenotypical Spectrum of PPCS-Related Disorders. AM. J. Med. Genet. A.

[40] Cavestro C., Diodato D., Tiranti V., Di Meo I. (2023). Inherited Disorders of Coenzyme A Biosynthesis: Models, Mechanisms, and Treatments. Int. J. Mol. Sci..

[41] Brezavar D., Bonnen P.E. (2019). Incidence of PKAN Determined by Bioinformatic and Population-Based Analysis of ~140,000 Humans. Mol. Genet. Metab..

[42] Zhou B., Westaway S.K., Levinson B., Johnson M.A., Gitschier J., Hayflick S.J. (2001). A Novel Pantothenate Kinase Gene (PANK2) Is Defective in Hallervorden-Spatz Syndrome. Nat. Genet..

[43] Wydrych A., Pakuła B., Janikiewicz J., Dobosz A.M., Jakubek-Olszewska P., Skowrońska M., Kurkowska-Jastrzębska I., Cwyl M., Popielarz M., Pinton P. (2025). Metabolic Impairments in Neurodegeneration with Brain Iron Accumulation. Biochim. Biophys. Acta (BBA) -Bioenerg..

[44] Rock C.O., Karim M.A., Zhang Y.-M., Jackowski S. (2002). The Murine Pantothenate Kinase (Pank1) Gene Encodes Two Differentially Regulated Pantothenate Kinase Isozymesq. Gene.

[45] Kotzbauer P.T., Truax A.C., Trojanowski J.Q., Lee V.M.-Y. (2005). Altered Neuronal Mitochondrial Coenzyme A Synthesis in Neurodegeneration with Brain Iron Accumulation Caused by Abnormal Processing, Stability, and Catalytic Activity of Mutant Pantothenate Kinase 2. J. Neurosci..

[46] Leonardi R., Rock C.O., Jackowski S., Zhang Y.-M. (2007). Activation of Human Mitochondrial Pantothenate Kinase 2 by Palmitoylcarnitine. Proc. Natl. Acad. Sci. USA.

[47] Reuter S.E., Evans A.M. (2012). Carnitine and Acylcarnitines: Pharmacokinetic, pharmacological and clinical aspects. Clin. Pharmacokinet..

[48] Ching K.H.L., Westaway S.K., Gitschier J., Higgins J.J., Hayflick S.J. (2002). HARP Syndrome Is Allelic with Pantothenate Kinase–Associated Neurodegeneration. Neurology.

[49] Hartig M.B., Hörtnagel K., Garavaglia B., Zorzi G., Kmiec T., Klopstock T., Rostasy K., Svetel M., Kostic V.S., Schuelke M. (2006). Genotypic and Phenotypic Spectrum of *PANK2* Mutations in Patients with Neurodegeneration with Brain Iron Accumulation. Ann. Neurol..

[50] Freeman K., Gregory A., Turner A., Blasco P., Hogarth P., Hayflick S. (2007). Intellectual and Adaptive Behaviour Functioning in Pantothenate Kinase-associated Neurodegeneration. J. Intellect. Disabil. Res..

[51] Pellecchia M.T., Valente E.M., Cif L., Salvi S., Albanese A., Scarano V., Bonuccelli U., Bentivoglio A.R., D’Amico A., Marelli C. (2005). The Diverse Phenotype and Genotype of Pantothenate Kinase-Associated Neurodegeneration. Neurology.

[52] Lee J.-H., Gregory A., Hogarth P., Rogers C., Hayflick S.J. (2018). Looking Deep into the Eye-of-the-Tiger in Pantothenate Kinase–Associated Neurodegeneration. AJNR AM. J. Neuroradiol..

[53] Santambrogio P., Ripamonti M., Paolizzi C., Panteghini C., Carecchio M., Chiapparini L., Raimondi M., Rubio A., Di Meo I., Cozzi A. (2020). Harmful Iron-Calcium Relationship in Pantothenate Kinase Associated Neurodegeneration. Int. J. Mol. Sci..

[54] Stoeter P., Roa-Sanchez P., Speckter H., Perez-Then E., Foerster B., Vilchez C., Oviedo J., Rodriguez-Raecke R. (2015). Changes of Cerebral White Matter in Patients Suffering from Pantothenate Kinase-Associated Neurodegeneration (PKAN): A Diffusion Tensor Imaging (DTI) Study. Park. Relat. Disord..

[55] Zizioli D., Tiso N., Guglielmi A., Saraceno C., Busolin G., Giuliani R., Khatri D., Monti E., Borsani G., Argenton F. (2016). Knock-down of Pantothenate Kinase 2 Severely Affects the Development of the Nervous and Vascular System in Zebrafish, Providing New Insights into PKAN Disease. Neurobiol. Dis..

[56] Mignani L., Zizioli D., Khatri D., Facchinello N., Schiavone M., De Palma G., Finazzi D. (2022). Bi-Allelic Mutations in Zebrafish Pank2 Gene Lead to Testicular Atrophy and Perturbed Behavior without Signs of Neurodegeneration. Int. J. Mol. Sci..

[57] Varun P., Hagit T., Uriya B., Sagiv S., Sebastian K. (2013). A New in Vivo Model of Pantothenate Kinase-Associated Neurodegeneration Reveals a Surprising Role for Transcriptional Regulation in Pathogenesis. Front. Cell. Neurosci..

[58] Kuo Y.-M., Duncan J.L., Westaway S.K., Yang H., Nune G., Xu E.Y., Hayflick S.J., Gitschier J. (2005). Deficiency of Pantothenate Kinase 2 (Pank2) in Mice Leads to Retinal Degeneration and Azoospermia. Hum. Mol. Genet..

[59] Brunetti D., Dusi S., Morbin M., Uggetti A., Moda F., D’Amato I., Giordano C., d’Amati G., Cozzi A., Levi S. (2012). Pantothenate Kinase-Associated Neurodegeneration: Altered Mitochondria Membrane Potential and Defective Respiration in Pank2 Knock-out Mouse Model. Hum. Mol. Genet..

[60] Brunetti D., Dusi S., Giordano C., Lamperti C., Morbin M., Fugnanesi V., Marchet S., Fagiolari G., Sibon O., Moggio M. (2014). Pantethine Treatment Is Effective in Recovering the Disease Phenotype Induced by Ketogenic Diet in a Pantothenate Kinase-Associated Neurodegeneration Mouse Model. Brain.

[61] Orellana D.I., Santambrogio P., Rubio A., Yekhlef L., Cancellieri C., Dusi S., Giannelli S.G., Venco P., Mazzara P.G., Cozzi A. (2016). Coenzyme A Corrects Pathological Defects in Human Neurons of PANK 2-associated Neurodegeneration. EMBO Mol. Med..

[62] Santambrogio P., Ripamonti M., Cozzi A., Raimondi M., Cavestro C., Di Meo I., Rubio A., Taverna S., Tiranti V., Levi S. (2022). Massive Iron Accumulation in PKAN-Derived Neurons and Astrocytes: Light on the Human Pathological Phenotype. Cell Death Dis..

[63] Ripamonti M., Santambrogio P., Racchetti G., Cozzi A., Di Meo I., Tiranti V., Levi S. (2022). PKAN hiPS-Derived Astrocytes Show Impairment of Endosomal Trafficking: A Potential Mechanism Underlying Iron Accumulation. Front. Cell. Neurosci..

[64] Leoni V., Strittmatter L., Zorzi G., Zibordi F., Dusi S., Garavaglia B., Venco P., Caccia C., Souza A.L., Deik A. (2012). Metabolic Consequences of Mitochondrial Coenzyme A Deficiency in Patients with PANK2 Mutations. Mol. Genet. Metab..

[65] Zhyvoloup A., Nemazanyy I., Babich A., Panasyuk G., Pobigailo N., Vudmaska M., Naidenov V., Kukharenko O., Palchevskii S., Savinska L. (2002). Molecular Cloning of CoA Synthase. J. Biol. Chem..

[66] Leonardi R., Zhang Y.-M., Rock C.O., Jackowski S. (2005). Coenzyme A: Back in Action. Prog. Lipid Res..

[67] Mishra R., Kulshreshtha S., Mandal K., Khurana A., Diego-Álvarez D., Pradas L., Saxena R., Phadke S., Moirangthem A., Masih S. (2022). *COASY* Related Pontocerebellar Hypoplasia Type 12: A Common Indian Mutation with Expansion of the Phenotypic Spectrum. Am. J. Med. Genet. Part A.

[68] Rosati J., Johnson J., Stander Z., White A., Tortorelli S., Bailey D., Fong C., Lee B.H. (2023). Progressive Brain Atrophy and Severe Neurodevelopmental Phenotype in Siblings with Biallelic *COASY* Variants. Am. J. Med. Genet. Part A.

[69] Evers C., Seitz A., Assmann B., Opladen T., Karch S., Hinderhofer K., Granzow M., Paramasivam N., Eils R., Diessl N. (2017). Diagnosis of CoPAN by Whole Exome Sequencing: Waking up a Sleeping Tiger’s Eye. Am. J. Med. Genet. Part A.

[70] Ceccatelli Berti C., Dallabona C., Lazzaretti M., Dusi S., Tosi E., Tiranti V., Goffrini P. (2015). Modeling Human Coenzyme A Synthase Mutation in Yeast Reveals Altered Mitochondrial Function, Lipid Content and Iron Metabolism. Microb. Cell.

[71] Khatri D., Zizioli D., Tiso N., Facchinello N., Vezzoli S., Gianoncelli A., Memo M., Monti E., Borsani G., Finazzi D. (2016). Down-Regulation of Coasy, the Gene Associated with NBIA-VI, Reduces Bmp Signaling, Perturbs Dorso-Ventral Patterning and Alters Neuronal Development in Zebrafish. Sci. Rep..

[72] Cavestro C., D’Amato M., Colombo M.N., Cascone F., Moro A.S., Levi S., Tiranti V., Di Meo I. (2024). CoA Synthase Plays a Critical Role in Neurodevelopment and Neurodegeneration. Front. Cell. Neurosci..

[73] Di Meo I., Cavestro C., Pedretti S., Fu T., Ligorio S., Manocchio A., Lavermicocca L., Santambrogio P., Ripamonti M., Levi S. (2020). Neuronal Ablation of CoA Synthase Causes Motor Deficits, Iron Dyshomeostasis, and Mitochondrial Dysfunctions in a CoPAN Mouse Model. Int. J. Mol. Sci..

[74] Cavestro C., Cascone F., Legati A., Izzo R., Catania M., Vergara C., Rodríguez-Pascau L., Pizcueta P., Tiranti V., Di Meo I. (2026). PPARγ Activation by Leriglitazone Counteracts Neurodegeneration and Neuroinflammation in a Disease-Relevant Mouse Model of COASY Dysfunction. Pharmacol. Res..

[75] Guo Y.-P., Tang B.-S., Guo J.-F. (2018). PLA2G6-Associated Neurodegeneration (PLAN): Review of Clinical Phenotypes and Genotypes. Front. Neurol..

[76] Winstead M.V., Balsinde J., Dennis E.A. (2000). Calcium-Independent Phospholipase A2: Structure and Function. Biochim. Biophys. Acta (BBA) -Mol. Cell Biol. Lipids.

[77] Chu Y.-T., Lin H.-Y., Chen P.-L., Lin C.-H. (2020). Genotype-Phenotype Correlations of Adult-Onset PLA2G6-Associated Neurodegeneration: Case Series and Literature Review. BMC Neurol..

[78] Hayashi D., Dennis E.A. (2023). Molecular Basis of Unique Specificity and Regulation of Group VIA Calcium-Independent Phospholipase A2 (PNPLA9) and Its Role in Neurodegenerative Diseases. Pharmacol. Ther..

[79] Burke J.E., Dennis E.A. (2009). Phospholipase A2 Biochemistry. Cardiovasc. Drugs Ther..

[80] Gregory A., Kurian M.A., Soo A.K., Wilson J.L., Hogarth P., Hayflick S.J., Adam M.P., Bick S., Mirzaa G.M., Pagon R.A., Wallace S.E., Amemiya A. (1993). PLA2G6-Associated Neurodegeneration. GeneReviews^®^.

[81] Nardocci N., Zorzi G., Farina L., Binelli S., Scaioli W., Ciano C., Verga L., Angelini L., Savoiardo M., Bugiani O. (1999). Infantile Neuroaxonal Dystrophy: Clinical Spectrum and Diagnostic Criteria. Neurology.

[82] Fusco C., Frattini D., Panteghini C., Pascarella R., Garavaglia B. (2015). A Case of Infantile Neuroaxonal Dystrophy of Neonatal Onset. J. Child. Neurol..

[83] Gregory A., Westaway S.K., Holm I.E., Kotzbauer P.T., Hogarth P., Sonek S., Coryell J.C., Nguyen T.M., Nardocci N., Zorzi G. (2008). Neurodegeneration Associated with Genetic Defects in Phospholipase A2. Neurology.

[84] Magrinelli F., Mehta S., Di Lazzaro G., Latorre A., Edwards M.J., Balint B., Basu P., Kobylecki C., Groppa S., Hegde A. (2022). Dissecting the Phenotype and Genotype of PLA2G6-Related Parkinsonism. Mov. Disord..

[85] Karkheiran S., Shahidi G.A., Walker R.H., Paisán-Ruiz C. (2015). PLA2G6-Associated Dystonia–Parkinsonism: Case Report and Literature Review. Tremor Other Hyperkinetic Mov..

[86] Schneider S.A., Dusek P., Hardy J., Westenberger A., Jankovic J., Bhatia K.P. (2013). Genetics and Pathophysiology of Neurodegeneration with Brain Iron Accumulation (NBIA). Curr. Neuropharmacol..

[87] Erskine D., Attems J. (2021). Insights into Lewy Body Disease from Rare Neurometabolic Disorders. J. Neural Transm..

[88] Kinghorn K.J., Castillo-Quan J.I., Bartolome F., Angelova P.R., Li L., Pope S., Cochemé H.M., Khan S., Asghari S., Bhatia K.P. (2015). Loss of *PLA2G6* Leads to Elevated Mitochondrial Lipid Peroxidation and Mitochondrial Dysfunction. Brain.

[89] Mori A., Hatano T., Inoshita T., Shiba-Fukushima K., Koinuma T., Meng H., Kubo S., Spratt S., Cui C., Yamashita C. (2019). Parkinson’s Disease-Associated *iPLA2-VIA/*PLA2G6 Regulates Neuronal Functions and α-Synuclein Stability through Membrane Remodeling. Proc. Natl. Acad. Sci. USA.

[90] Beck G., Shinzawa K., Hayakawa H., Baba K., Yasuda T., Sumi-Akamaru H., Tsujimoto Y., Mochizuki H. (2015). Deficiency of Calcium-Independent Phospholipase A2 Beta Induces Brain Iron Accumulation through Upregulation of Divalent Metal Transporter 1. PLoS ONE.

[91] Malik I., Turk J., Mancuso D.J., Montier L., Wohltmann M., Wozniak D.F., Schmidt R.E., Gross R.W., Kotzbauer P.T. (2008). Disrupted Membrane Homeostasis and Accumulation of Ubiquitinated Proteins in a Mouse Model of Infantile Neuroaxonal Dystrophy Caused by PLA2G6 Mutations. Am. J. Pathol..

[92] Shinzawa K., Sumi H., Ikawa M., Matsuoka Y., Okabe M., Sakoda S., Tsujimoto Y. (2008). Neuroaxonal Dystrophy Caused by Group VIA Phospholipase A2 Deficiency in Mice: A Model of Human Neurodegenerative Disease. J. Neurosci..

[93] Sumi-Akamaru H., Beck G., Shinzawa K., Kato S., Riku Y., Yoshida M., Fujimura H., Tsujimoto Y., Sakoda S., Mochizuki H. (2016). High Expression of α-Synuclein in Damaged Mitochondria with PLA2G6 Dysfunction. Acta Neuropathol. Commun..

[94] Villalón-García I., Álvarez-Córdoba M., Povea-Cabello S., Talaverón-Rey M., Villanueva-Paz M., Luzón-Hidalgo R., Suárez-Rivero J.M., Suárez-Carrillo A., Munuera-Cabeza M., Salas J.J. (2022). Vitamin E Prevents Lipid Peroxidation and Iron Accumulation in PLA2G6-Associated Neurodegeneration. Neurobiol. Dis..

[95] Ke M., Chong C.-M., Zeng H., Huang M., Huang Z., Zhang K., Cen X., Lu J.-H., Yao X., Qin D. (2020). Azoramide Protects iPSC-Derived Dopaminergic Neurons with PLA2G6 D331Y Mutation through Restoring ER Function and CREB Signaling. Cell Death Dis..

[96] Angelini C., Durand C.M., Fergelot P., Deforges J., Vital A., Menegon P., Sarrazin E., Bellance R., Mathis S., Gonzalez V. (2023). Autosomal Dominant MPAN: Mosaicism Expands the Clinical Spectrum to Atypical Late-Onset Phenotypes. Mov. Disord..

[97] Hartig M., Prokisch H., Meitinger T., Klopstock T. (2013). Mitochondrial Membrane Protein-Associated Neurodegeneration (MPAN). Int. Rev. Neurobiol..

[98] Klingelhuber F., Frendo-Cumbo S., Omar-Hmeadi M., Massier L., Kakimoto P., Taylor A.J., Couchet M., Ribicic S., Wabitsch M., Messias A.C. (2024). A Spatiotemporal Proteomic Map of Human Adipogenesis. Nat. Metab..

[99] Iuso A., Sibon O.C.M., Gorza M., Heim K., Organisti C., Meitinger T., Prokisch H. (2014). Impairment of Drosophila Orthologs of the Human Orphan Protein C19orf12 Induces Bang Sensitivity and Neurodegeneration. PLoS ONE.

[100] Mignani L., Zizioli D., Borsani G., Monti E., Finazzi D. (2020). The Downregulation of C19orf12 Negatively Affects Neuronal and Musculature Development in Zebrafish Embryos. Front. Cell Dev. Biol..

[101] Shao C., Zhu J., Ma X., Siedlak S.L., Cohen M.L., Lerner A., Wang W. (2022). C19orf12 Ablation Causes Ferroptosis in Mitochondrial Membrane Protein-Associated with Neurodegeneration. Free Radic. Biol. Med..

[102] Venco P., Bonora M., Giorgi C., Papaleo E., Iuso A., Prokisch H., Pinton P., Tiranti V. (2015). Mutations of C19orf12, Coding for a Transmembrane Glycine Zipper Containing Mitochondrial Protein, Cause Mis-Localization of the Protein, Inability to Respond to Oxidative Stress and Increased Mitochondrial Ca^2+^. Front. Genet..

[103] Zanuttigh E., Derderian K., Güra M.A., Geerlof A., Di Meo I., Cavestro C., Hempfling S., Ortiz-Collazos S., Mauthe M., Kmieć T. (2023). Identification of Autophagy as a Functional Target Suitable for the Pharmacological Treatment of Mitochondrial Membrane Protein-Associated Neurodegeneration (MPAN) In Vitro. Pharmaceutics.

[104] Papandreou A., Soo A.K.S., Spaull R., Mankad K., Kurian M.A., Sudhakar S. (2022). Expanding the Spectrum of Early Neuroradiologic Findings in β Propeller Protein-Associated Neurodegeneration. AJNR Am. J. Neuroradiol..

[105] Gregory A., Kurian M.A., Haack T., Hayflick S.J., Hogarth P., Adam M.P., Bick S., Mirzaa G.M., Pagon R.A., Wallace S.E., Amemiya A. (1993). Beta-Propeller Protein-Associated Neurodegeneration. GeneReviews^®^.

[106] Wilson J.L., Gregory A., Kurian M.A., Bushlin I., Mochel F., Emrick L., Adang L., Hogarth P., Hayflick S.J., BPAN Guideline Contributing Author Group (2021). Consensus Clinical Management Guideline for Beta-propeller Protein-associated Neurodegeneration. Dev. Med. Child. Neurol..

[107] Kimura Y., Sato N., Ishiyama A., Shigemoto Y., Suzuki F., Fujii H., Maikusa N., Matsuda H., Nishioka K., Hattori N. (2021). Serial MRI Alterations of Pediatric Patients with Beta-Propeller Protein Associated Neurodegeneration (BPAN). J. Neuroradiol..

[108] Fleming A., Bourdenx M., Fujimaki M., Karabiyik C., Krause G.J., Lopez A., Martín-Segura A., Puri C., Scrivo A., Skidmore J. (2022). The Different Autophagy Degradation Pathways and Neurodegeneration. Neuron.

[109] Noda M., Ito H., Nagata K. (2021). Physiological Significance of WDR45, a Responsible Gene for β-Propeller Protein Associated Neurodegeneration (BPAN), in Brain Development. Sci. Rep..

[110] Zhao Y.G., Sun L., Miao G., Ji C., Zhao H., Sun H., Miao L., Yoshii S.R., Mizushima N., Wang X. (2015). The Autophagy Gene Wdr45/Wipi4 Regulates Learning and Memory Function and Axonal Homeostasis. Autophagy.

[111] Wan H., Wang Q., Chen X., Zeng Q., Shao Y., Fang H., Liao X., Li H.-S., Liu M.-G., Xu T.-L. (2020). WDR45 Contributes to Neurodegeneration through Regulation of ER Homeostasis and Neuronal Death. Autophagy.

[112] Shimizu T., Tamura N., Nishimura T., Saito C., Yamamoto H., Mizushima N. (2023). Comprehensive Analysis of Autophagic Functions of WIPI Family Proteins and Their Implications for the Pathogenesis of β-Propeller Associated Neurodegeneration. Hum. Mol. Genet..

[113] Lu Q., Yang P., Huang X., Hu W., Guo B., Wu F., Lin L., Kovács A.L., Yu L., Zhang H. (2011). The WD40 Repeat PtdIns(3)P-Binding Protein EPG-6 Regulates Progression of Omegasomes to Autophagosomes. Dev. Cell.

[114] Lee J.-H., Nam S.O., Kim E.K., Shin J.-H., Oh S.H., Ryu D., Lee H.E., Mun J.Y. (2021). Autophagic Defects Observed in Fibroblasts from a Patient with β-Propeller Protein-Associated Neurodegeneration. Am. J. Med. Genet. Part A.

[115] Seibler P., Burbulla L.F., Dulovic M., Zittel S., Heine J., Schmidt T., Rudolph F., Westenberger A., Rakovic A., Münchau A. (2018). Iron Overload Is Accompanied by Mitochondrial and Lysosomal Dysfunction in WDR45 Mutant Cells. Brain.

[116] Saitsu H., Nishimura T., Muramatsu K., Kodera H., Kumada S., Sugai K., Kasai-Yoshida E., Sawaura N., Nishida H., Hoshino A. (2013). De Novo Mutations in the Autophagy Gene WDR45 Cause Static Encephalopathy of Childhood with Neurodegeneration in Adulthood. Nat. Genet..

[117] Aring L., Choi E.-K., Kopera H., Lanigan T., Iwase S., Klionsky D.J., Seo Y.A.J. (2022). A neurodegeneration gene, *WDR45*, links impaired ferritinophagy to iron accumulation. Neurochem.

[118] Ceraolo G., Spoto G., Consoli C., Modafferi E., Di Rosa G., Nicotera A.G. (2025). Pediatric Genetic Dystonias: Current Diagnostic Approaches and Treatment Options. Life.

[119] Hong G., Zhang Z., Wang P., Li G., Zhang W., Zou H., Luo X. (2024). Case Report: Asymmetric Bilateral Deep Brain Stimulation for the Treatment of Pantothenate Kinase-Associated Neurodegeneration in a Patient: A Unique Case of Atypical PKAN with a Novel Heterozygous PANK2 Mutation. Front. Hum. Neurosci..

[120] Levi V., Stanziano M., Pinto C., Zibordi F., Fedeli D., Caldiera V., Cilia R., Golfrè Andreasi N., Braccia A., Carozzi C. (2024). Bilateral Simultaneous Magnetic Resonance–Guided Focused Ultrasound Pallidotomy for Life-Threatening Status Dystonicus. Mov. Disord..

[121] Iankova V., Karin I., Klopstock T., Schneider S.A. (2021). Emerging Disease-Modifying Therapies in Neurodegeneration with Brain Iron Accumulation (NBIA) Disorders. Front. Neurol..

[122] Klopstock T., Tricta F., Neumayr L., Karin I., Zorzi G., Fradette C., Kmieć T., Büchner B., Steele H.E., Horvath R. (2019). Safety and Efficacy of Deferiprone for Pantothenate Kinase-Associated Neurodegeneration: A Randomised, Double-Blind, Controlled Trial and an Open-Label Extension Study. Lancet Neurol..

[123] Chen S., Lai X., Fu J., Yang J., Zhao B., Shang H., Huang R., Chen X. (2023). A Novel C19ORF12 Mutation in Two MPAN Sisters Treated with Deferiprone. BMC Neurol..

[124] Fonderico M., Laudisi M., Andreasi N.G., Bigoni S., Lamperti C., Panteghini C., Garavaglia B., Carecchio M., Emanuele E.A., Forni G.L. (2017). Patient Affected by Beta-Propeller Protein-Associated Neurodegeneration: A Therapeutic Attempt with Iron Chelation Therapy. Front. Neurol..

[125] Lim S.-Y., Tan A.H., Ahmad-Annuar A., Schneider S.A., Bee P.C., Lim J.L., Ramli N., Idris M.I. (2018). A Patient with Beta-Propeller Protein-Associated Neurodegeneration: Treatment with Iron Chelation Therapy. J. Mov. Disord..

[126] Marupudi N., Xiong M.P. (2024). Genetic Targets and Applications of Iron Chelators for Neurodegeneration with Brain Iron Accumulation. ACS Bio Med. Chem. Au.

[127] Hanson L.R., Fine J.M., Renner D.B., Svitak A.L., Burns R.B., Nguyen T.M., Tuttle N.J., Marti D.L., Panter S.S., Frey W.H. (2012). Intranasal Delivery of Deferoxamine Reduces Spatial Memory Loss in APP/PS1 Mice. Drug Deliv. Transl. Res..

[128] Hanson L.R., Roeytenberg A., Martinez P.M., Coppes V.G., Sweet D.C., Rao R.J., Marti D.L., Hoekman J.D., Matthews R.B., Frey W.H. (2009). Intranasal Deferoxamine Provides Increased Brain Exposure and Significant Protection in Rat Ischemic Stroke. J. Pharmacol. Exp. Ther..

[129] Mähler A., Mandel S., Lorenz M., Ruegg U., Wanker E.E., Boschmann M., Paul F. (2013). Epigallocatechin-3-Gallate: A Useful, Effective and Safe Clinical Approach for Targeted Prevention and Individualised Treatment of Neurological Diseases?. EPMA J..

[130] Zou Z., Shao S., Zou R., Qi J., Chen L., Zhang H., Shen Q., Yang Y., Ma L., Guo R. (2019). Linking the Low-density Lipoprotein Receptor-binding Segment Enables the Therapeutic 5-YHEDA Peptide to Cross the Blood-brain Barrier and Scavenge Excess Iron and Radicals in the Brain of Senescent Mice. Alzheimer’s Dement. Transl. Res. Clin. Interv..

[131] Ma J., Liu J., Chen S., Zhang W., Wang T., Cao M., Yang Y., Du Y., Cui G., Du Z. (2024). Understanding the Mechanism of Ferroptosis in Neurodegenerative Diseases. Front. Biosci. (Landmark Ed.).

[132] Bhowmick S., Lee Y.J. (2025). ER Stress Induced by Artemisinin and Its Derivatives Determines the Susceptibility to Their Synergistic Apoptotic Killing With TRAIL. Cancer Med..

[133] Schmuck G., Roehrdanz E., Haynes R.K., Kahl R. (2002). Neurotoxic Mode of Action of Artemisinin. Antimicrob. Agents Chemother..

[134] Srinivasan B., Baratashvili M., Van Der Zwaag M., Kanon B., Colombelli C., Lambrechts R.A., Schaap O., Nollen E.A., Podgoršek A., Kosec G. (2015). Extracellular 4′-Phosphopantetheine Is a Source for Intracellular Coenzyme A Synthesis. Nat. Chem. Biol..

[135] Khatri D., Zizioli D., Trivedi A., Borsani G., Monti E., Finazzi D. (2019). Overexpression of Human Mutant PANK2 Proteins Affects Development and Motor Behavior of Zebrafish Embryos. Neuromol. Med..

[136] Rana A., Seinen E., Siudeja K., Muntendam R., Srinivasan B., van der Want J.J., Hayflick S., Reijngoud D.-J., Kayser O., Sibon O.C.M. (2010). Pantethine Rescues a Drosophila Model for Pantothenate Kinase–Associated Neurodegeneration. Proc. Natl. Acad. Sci. USA.

[137] Jeong S.Y., Hogarth P., Placzek A., Gregory A.M., Fox R., Zhen D., Hamada J., van der Zwaag M., Lambrechts R., Jin H. (2019). 4′-Phosphopantetheine Corrects CoA, Iron, and Dopamine Metabolic Defects in Mammalian Models of PKAN. EMBO Mol. Med..

[138] Di Meo I., Colombelli C., Srinivasan B., De Villiers M., Hamada J., Jeong S.Y., Fox R., Woltjer R.L., Tepper P.G., Lahaye L.L. (2017). Acetyl-4′-Phosphopantetheine Is Stable in Serum and Prevents Phenotypes Induced by Pantothenate Kinase Deficiency. Sci. Rep..

[139] Klopstock T., Videnovic A., Bischoff A.T., Bonnet C., Cif L., Comella C., Correa-Vela M., Escolar M.L., Fraser J.L., Gonzalez V. (2021). Fosmetpantotenate Randomized Controlled Trial in Pantothenate Kinase–Associated Neurodegeneration. Mov. Disord..

[140] Bracke M.M.G., Polet S.S., Plantinga M., De Koning T.J. (2025). Mitigating the Impact of Study-Start Delays in Clinical Trials for Rare Disorders: Insights and Lessons from a PKAN Trial. Orphanet J. Rare Dis..

[141] Sharma L.K., Subramanian C., Yun M.-K., Frank M.W., White S.W., Rock C.O., Lee R.E., Jackowski S. (2018). A Therapeutic Approach to Pantothenate Kinase Associated Neurodegeneration. Nat. Commun..

[142] Subramanian C., Frank M.W., Sukhun R., Henry C.E., Wade A., Harden M.E., Rao S., Tangallapally R., Yun M.-K., White S.W. (2024). Pantothenate Kinase Activation Restores Brain Coenzyme A in a Mouse Model of Pantothenate Kinase-Associated Neurodegeneration. J. Pharmacol. Exp. Ther..

[143] Coker A.L., Tangallapally R., Yun M.-K., Subramanian C., Jayasinghe T., Miller K., Edwards A., Frank M., Jackowski S., Rock C.O. (2026). Discovery of Sulfonamide Pantothenate Kinase Activators and Elucidation of the Role of Isoform Selectivity in Cellular Pantothenate Kinase Activation. J. Med. Chem..

[144] Pizcueta P., Vergara C., Emanuele M., Vilalta A., Rodríguez-Pascau L., Martinell M. (2023). Development of PPARγ Agonists for the Treatment of Neuroinflammatory and Neurodegenerative Diseases: Leriglitazone as a Promising Candidate. Int. J. Mol. Sci..

[145] Santambrogio P., Cozzi A., Meo I.D., Cavestro C., Vergara C., Rodríguez-Pascau L., Martinell M., Pizcueta P., Tiranti V., Levi S. (2023). PPAR Gamma Agonist Leriglitazone Recovers Alterations Due to Pank2-Deficiency in hiPS-Derived Astrocytes. Pharmaceutics.

[146] Myint S.M.M.P., Sun L.Y. (2023). L-Serine: Neurological Implications and Therapeutic Potential. Biomedicines.

[147] Ye L., Sun Y., Jiang Z., Wang G. (2021). L-Serine, an Endogenous Amino Acid, Is a Potential Neuroprotective Agent for Neurological Disease and Injury. Front. Mol. Neurosci..

[148] Dunlop R.A., Carney J.M. (2021). Mechanisms of L-Serine-Mediated Neuroprotection Include Selective Activation of Lysosomal Cathepsins B and L. Neurotox. Res..

[149] Levine T.D., Miller R.G., Bradley W.G., Moore D.H., Saperstein D.S., Flynn L.E., Katz J.S., Forshew D.A., Metcalf J.S., Banack S.A. (2017). Phase I Clinical Trial of Safety of L-Serine for ALS Patients. Amyotroph. Lateral Scler. Front. Degener..

[150] Garofalo K., Penno A., Schmidt B.P., Lee H.-J., Frosch M.P., von Eckardstein A., Brown R.H., Hornemann T., Eichler F.S. (2011). Oral L-Serine Supplementation Reduces Production of Neurotoxic Deoxysphingolipids in Mice and Humans with Hereditary Sensory Autonomic Neuropathy Type 1. J. Clin. Investig..

[151] Lee H.E., Jung M., Choi K., Jang J.H., Hwang S.-K., Chae S., Lee J.-H., Mun J.Y. (2024). L-Serine Restored Lysosomal Failure in Cells Derived from Patients with BPAN Reducing Iron Accumulation with Eliminating Lipofuscin. Free Radic. Biol. Med..

[152] Talaverón-Rey M., Álvarez-Córdoba M., Villalón-García I., Povea-Cabello S., Suárez-Rivero J.M., Gómez-Fernández D., Romero-González A., Suárez-Carrillo A., Munuera-Cabeza M., Cilleros-Holgado P. (2023). Alpha-Lipoic Acid Supplementation Corrects Pathological Alterations in Cellular Models of Pantothenate Kinase-Associated Neurodegeneration with Residual PANK2 Expression Levels. Orphanet J. Rare Dis..

[153] Camiolo G., Tibullo D., Giallongo C., Romano A., Parrinello N.L., Musumeci G., Di Rosa M., Vicario N., Brundo M.V., Amenta F. (2019). α-Lipoic Acid Reduces Iron-Induced Toxicity and Oxidative Stress in a Model of Iron Overload. Int. J. Mol. Sci..

[154] Firsov A.M., Fomich M.A., Bekish A.V., Sharko O.L., Kotova E.A., Saal H.J., Vidovic D., Shmanai V.V., Pratt D.A., Antonenko Y.N. (2019). Threshold Protective Effect of Deuterated Polyunsaturated Fatty Acids on Peroxidation of Lipid Bilayers. FEBS J..

[155] Kinghorn K.J., Castillo-Quan J.I. (2016). Mitochondrial Dysfunction and Defects in Lipid Homeostasis as Therapeutic Targets in Neurodegeneration with Brain Iron Accumulation. Rare Dis..

[156] Adams D., Midei M., Dastgir J., Flora C., Molinari R.J., Heerinckx F., Endemann S., Atwal P., Milner P., Shchepinov M.S. (2020). Treatment of Infantile Neuroaxonal Dystrophy with RT001: A Di-Deuterated Ethyl Ester of Linoleic Acid: Report of Two Cases. JIMD Rep..

[157] Pereira A., Fischinger Moura de Souza C., Álvarez-Córdoba M., Reche-López D., Sánchez-Alcázar J.A. (2024). A Therapeutic Approach to Pantothenate Kinase Associated Neurodegeneration: A Pilot Study. Orphanet J. Rare Dis..

[158] Álvarez-Córdoba M., Reche-López D., Cilleros-Holgado P., Talaverón-Rey M., Villalón-García I., Povea-Cabello S., Suárez-Rivero J.M., Suárez-Carrillo A., Munuera-Cabeza M., Piñero-Pérez R. (2022). Therapeutic Approach with Commercial Supplements for Pantothenate Kinase-Associated Neurodegeneration with Residual PANK2 Expression Levels. Orphanet J. Rare Dis..

[159] Suárez-Carrillo A., Álvarez-Córdoba M., Romero-González A., Talaverón-Rey M., Povea-Cabello S., Cilleros-Holgado P., Piñero-Pérez R., Reche-López D., Gómez-Fernández D., Romero-Domínguez J.M. (2023). Antioxidants Prevent Iron Accumulation and Lipid Peroxidation, but Do Not Correct Autophagy Dysfunction or Mitochondrial Bioenergetics in Cellular Models of BPAN. Int. J. Mol. Sci..

[160] Aldini G., Altomare A., Baron G., Vistoli G., Carini M., Borsani L., Sergio F. (2018). N-Acetylcysteine as an Antioxidant and Disulphide Breaking Agent: The Reasons Why. Free Radic. Res..

[161] Rahim A.A., Kurian M.A., Zhou H., Ferguson R., Tabrizi S.J., Lignani G., Aquilina K., Waddington S.N. (2026). Genetic Therapies for Neurological Diseases. Pharmacol. Rev..

[162] Naldini L. (2015). Gene Therapy Returns to Centre Stage. Nature.

[163] Scheller E.L., Krebsbach P.H. (2009). Gene Therapy: Design and Prospects for Craniofacial Regeneration. J. Dent. Res..

[164] Bulcha J.T., Wang Y., Ma H., Tai P.W.L., Gao G. (2021). Viral Vector Platforms within the Gene Therapy Landscape. Signal Transduct. Target. Ther..

[165] Lin G., Tepe B., McGrane G., Tipon R.C., Croft G., Panwala L., Hope A., Liang A.J., Zuo Z., Byeon S.K. (2023). Exploring Therapeutic Strategies for Infantile Neuronal Axonal Dystrophy (INAD/PARK14). eLife.

[166] Hordeaux J., Wang Q., Katz N., Buza E.L., Bell P., Wilson J.M. (2018). The Neurotropic Properties of AAV-PHP.B Are Limited to C57BL/6J Mice. Mol. Ther..

[167] Carisi M.C., Shamber C., Bishop M., Sangster M., Chandrachud U., Meyerink B., Pilaz L.J., Grishchuk Y. (2025). AAV-Mediated Gene Transfer of WDR45 Corrects Neurological Deficits in the Mouse Model of Beta-Propeller Protein-Associated Neurodegeneration. Hum. Gene Ther..

[168] Bisi N., Pinzi L., Rastelli G., Tonali N. (2024). Early Diagnosis of Neurodegenerative Diseases: What Has Been Undertaken to Promote the Transition from PET to Fluorescence Tracers. Molecules.

[169] Selvam S., Ayyavoo V. (2024). Biomarkers in Neurodegenerative Diseases: A Broad Overview. Explor. Neuroprot. Ther..

[170] Hansson O. (2021). Biomarkers for Neurodegenerative Diseases. Nat. Med..

[171] Chang X., Zhang J., Jiang Y., Wang J., Wu Y. (2020). Natural History and Genotype-phenotype Correlation of Pantothenate Kinase-associated Neurodegeneration. CNS Neurosci. Ther..

[172] Saffari A., Schröter J., Garbade S.F., Alecu J.E., Ebrahimi-Fakhari D., Hoffmann G.F., Kölker S., Ries M., Syrbe S. (2022). Quantitative Retrospective Natural History Modeling of *WDR45* -Related Developmental and Epileptic Encephalopathy—A Systematic Cross-Sectional Analysis of 160 Published Cases. Autophagy.

[173] Gavazzi F., Pierce S.R., Vithayathil J., Cunningham K., Anderson K., McCann J., Moll A., Muirhead K., Sherbini O., Prange E. (2022). Psychometric Outcome Measures in Beta-Propeller Protein-Associated Neurodegeneration (BPAN). Mol. Genet. Metab..

[174] Gregory A., Anderson K.M., Loftus H., Baudier R.L., Wilson J.L., Hogarth P., Hayflick S.J. (2026). Prospective 5-year Natural History Study of Infantile *PLA2G6*-associated Neurodegeneration. Dev. Med. Child. Neurol..

[175] Uygun Ö., Özcan A., Aras F.K., Bozdemir E., Uğur İşeri S., Kırımtay K., Karabay A., Gültekin M., Akçakaya N.H., Mammadov O. (2025). Quantitative Iron Measurements in the Basal Ganglia of NBIA Patients Using QSM: Insights from a Tertiary Center. Ann. Clin. Transl. Neurol..

[176] Santomauro D.F., Miller P.A., Shadid J., Wulf Hanson S., Vo A., Roy D.J., Hagins H., Mantilla Herrera A.M., Scott J.G., Erskine H.E. (2026). Updated Trends in the Global Prevalence and Burden of Mental Disorders, 1990–2023: A Systematic Analysis for the Global Burden of Disease Study 2023. Lancet.

[177] Hou X., Zaks T., Langer R., Dong Y. (2021). Lipid Nanoparticles for mRNA Delivery. Nat. Rev. Mater..

[178] Reche-López D., Romero-González A., Álvarez-Córdoba M., Suárez-Carrillo A., Cilleros-Holgado P., Piñero-Pérez R., Gómez-Fernández D., Romero-Domínguez J.M., López-Cabrera A., González-Granero S. (2025). Biotin Induces Inactive Chromosome X Reactivation and Corrects Physiopathological Alterations in Beta-Propeller-Protein-Associated Neurodegeneration. Int. J. Mol. Sci..

[179] Zeng H., Daniel T.C., Lingineni A., Chee K., Talloo K., Gao X. (2024). Recent Advances in Prime Editing Technologies and Their Promises for Therapeutic Applications. Curr. Opin. Biotechnol..

[180] Spaull R.V.V., Soo A.K.S., Hogarth P., Hayflick S.J., Kurian M.A. (2021). Towards Precision Therapies for Inherited Disorders of Neurodegeneration with Brain Iron Accumulation. Tremor Other Hyperkinetic Mov..

